# Supplementary material for: Data‐driven discovery of gene expression markers distinguishing pediatric acute lymphoblastic leukemia subtypes
Source: Mol Oncol. 2025 Aug 11;19(12):3548–77. doi: 10.1002/1878-0261.70046 (PMC12688183; doi:10.1002/1878-0261.70046)
Supplement: Supplementary file 2 — Fig. S2. Distribution of age at leukemia diagnosis of 88 patients of a Danish cohort. [file MOL2-19-3548-s017.pdf]

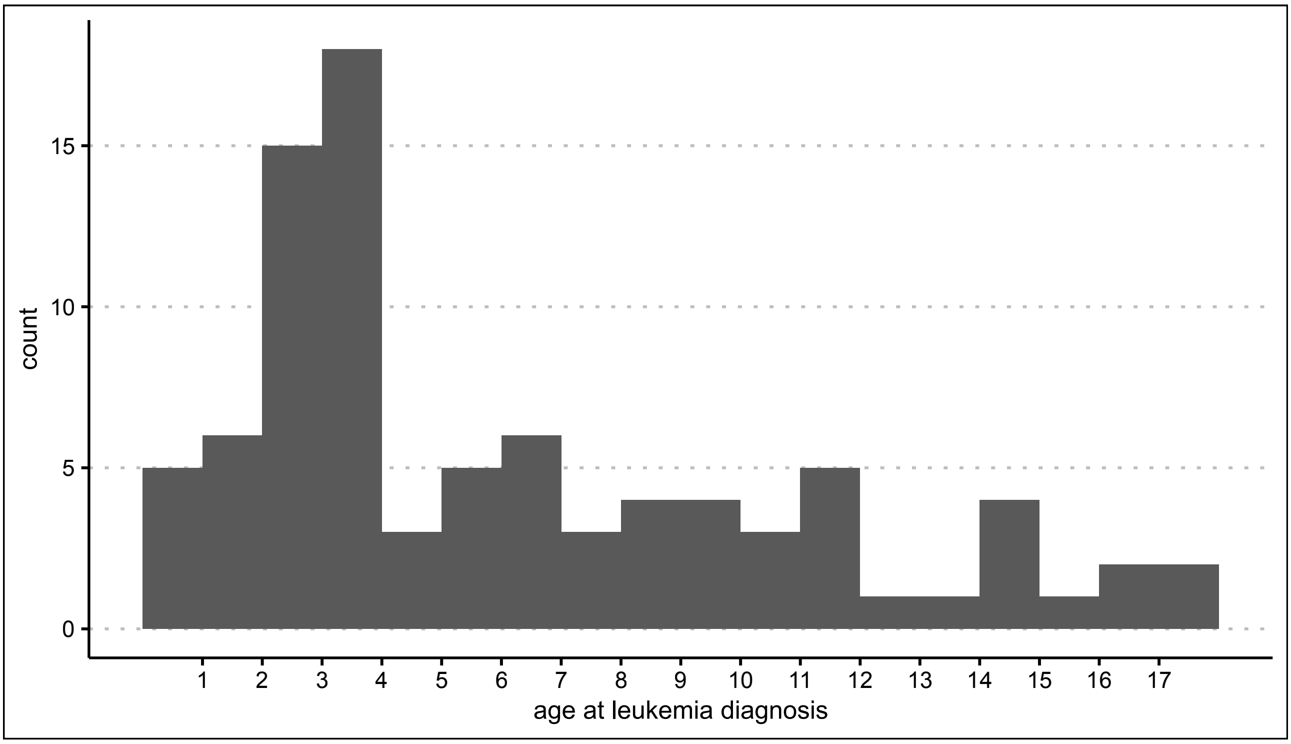

Supplementary Figure S2. Distribution of age at leukemia diagnosis of 88 patients of a Danish cohort.
