# Supplementary material for: Data‐driven discovery of gene expression markers distinguishing pediatric acute lymphoblastic leukemia subtypes
Source: Mol Oncol. 2025 Aug 11;19(12):3548–77. doi: 10.1002/1878-0261.70046 (PMC12688183; doi:10.1002/1878-0261.70046)
Supplement: Supplementary file 3 — Fig. S3. Density plot of log2 fold change values of 103 housekeeping consensus differentially expressed genes. [file MOL2-19-3548-s009.pdf]

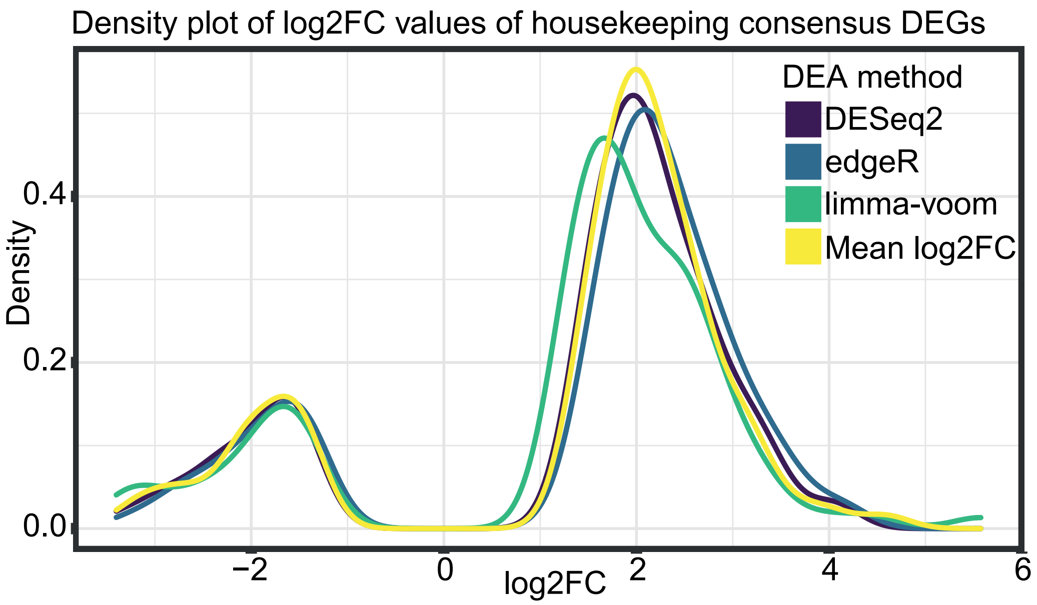

Supplementary Figure S3. Density plot of log2 fold change (log2FC) values of 103 housekeeping consensus differentially expressed genes (DEGs) predicted by three differential expression analysis (DEA) methods: limma-voom (green), edgeR (blue), and DESeq2 (purple). The distribution of the mean log2FC of the housekeeping DEGs across the three DEA methods is also shown (yellow). Housekeeping consensus DEGs were found by comparing the consensus DEGs with housekeeping genes reported by Eisenberg and Levanon (2013).
