# Supplementary material for: Data‐driven discovery of gene expression markers distinguishing pediatric acute lymphoblastic leukemia subtypes
Source: Mol Oncol. 2025 Aug 11;19(12):3548–77. doi: 10.1002/1878-0261.70046 (PMC12688183; doi:10.1002/1878-0261.70046)
Supplement: Supplementary file 4 — Fig. S4. Scree plot of percentage of explained variance for the first 20 principal component dimensions from principal component analysis. [file MOL2-19-3548-s013.pdf]

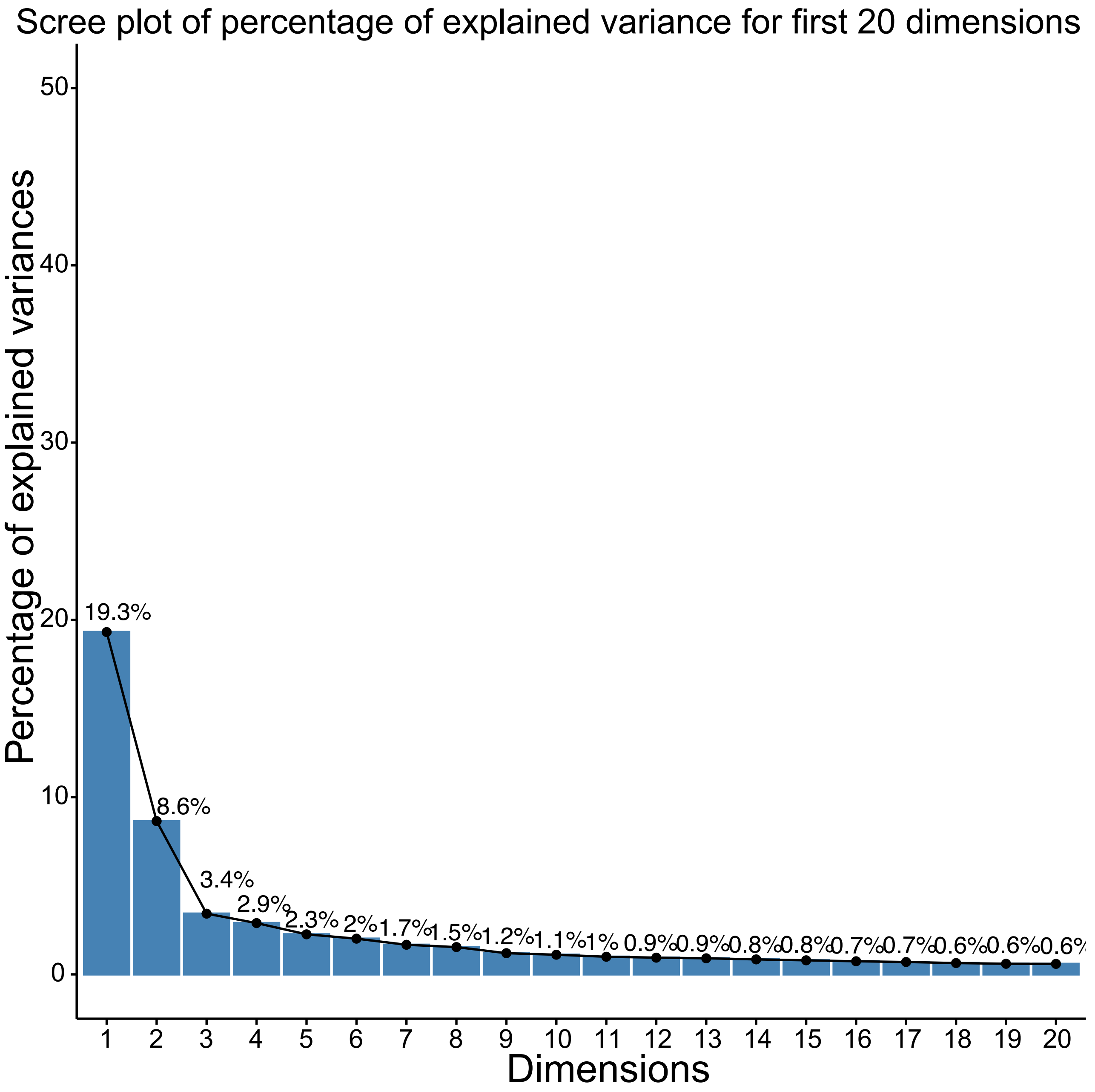

Supplementary Figure S4. Scree plot of percentage of explained variance for the first 20 principal component (PC) dimensions from principal component analysis (PCA). The percentage of explained variance for each of the first 20 dimensions are shown on top of each bar.
