# Supplementary material for: Data‐driven discovery of gene expression markers distinguishing pediatric acute lymphoblastic leukemia subtypes
Source: Mol Oncol. 2025 Aug 11;19(12):3548–77. doi: 10.1002/1878-0261.70046 (PMC12688183; doi:10.1002/1878-0261.70046)
Supplement: Supplementary file 5 — Fig. S5. Kaplan–Meier survival plots of nine of the discovered subtype‐related gene expression markers. [file MOL2-19-3548-s016.pdf]

A)

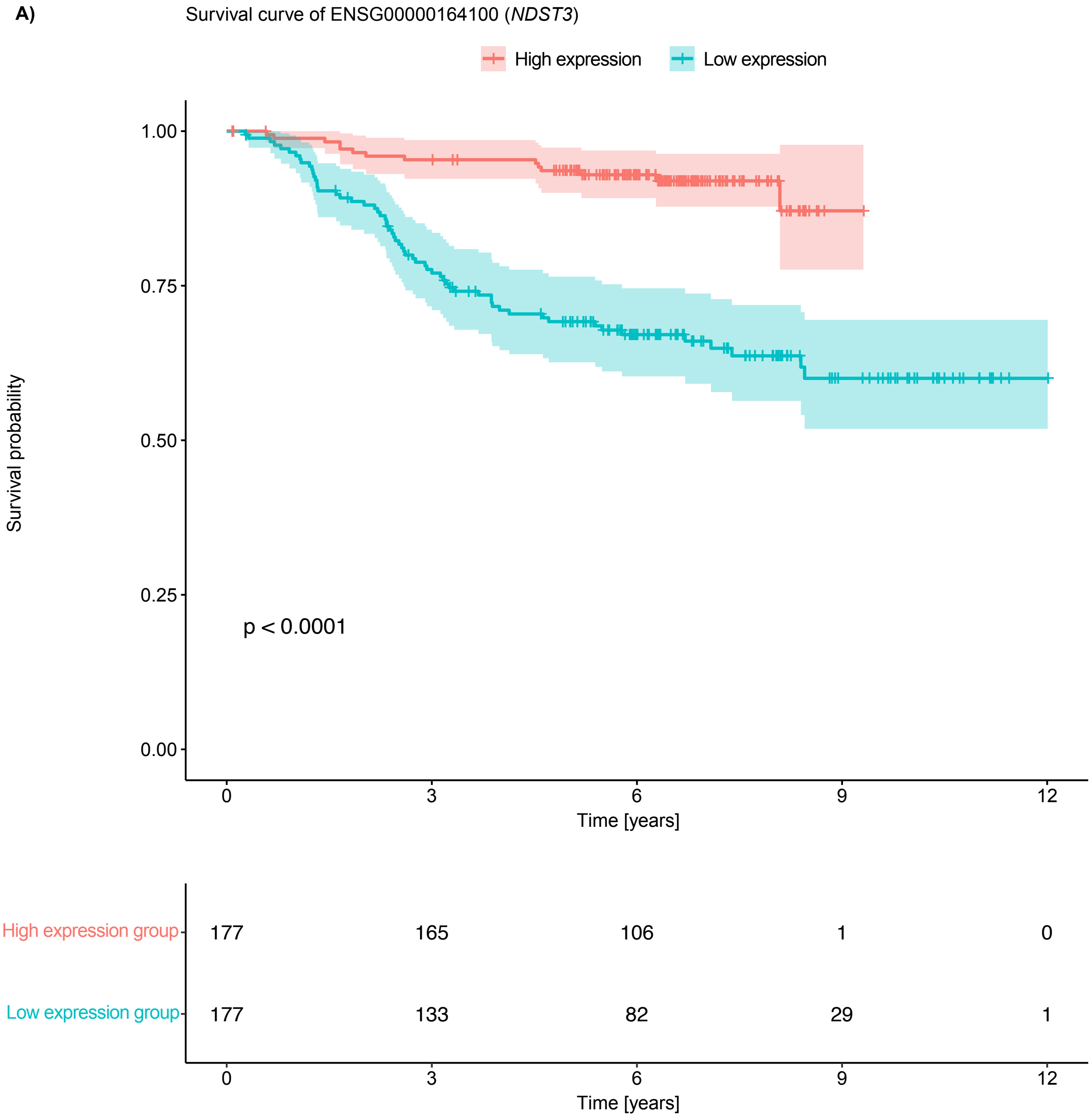

Survival curve of ENSG00000164100 (*NDST3*)
High expression
Low expression
1.00
0.75
0.50
0.25
0.00
Survival probability
p < 0.0001
0
3
6
9
12
Time [years]
High expression group
177
165
106
1
0
Low expression group
177
133
82
29
1
0
3
6
9
12
Time [years]

B) Survival curve of ENSG00000199683 (RN7SKP185)

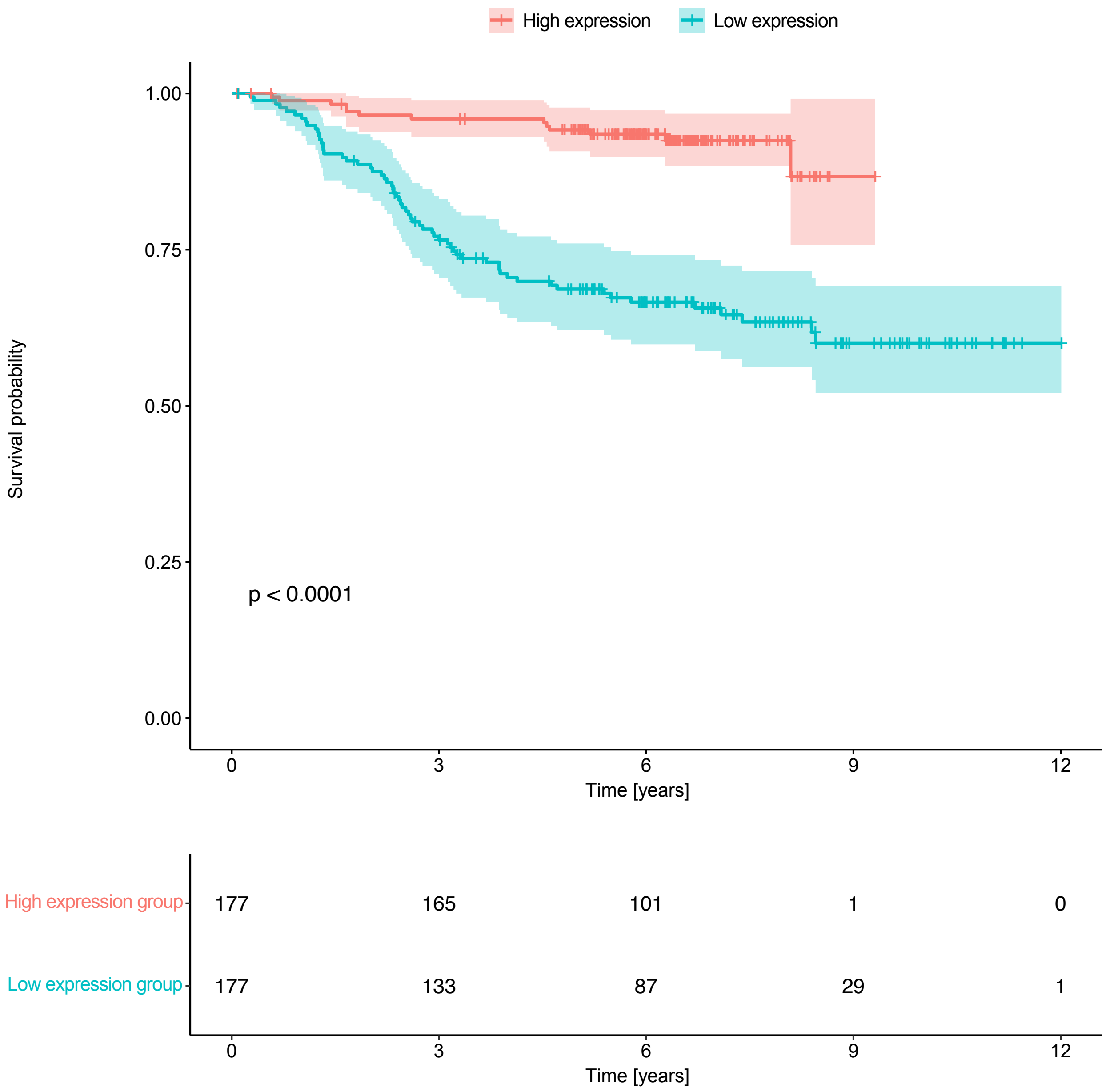

C) Survival curve of ENSG00000199831 (RN7SKP291)

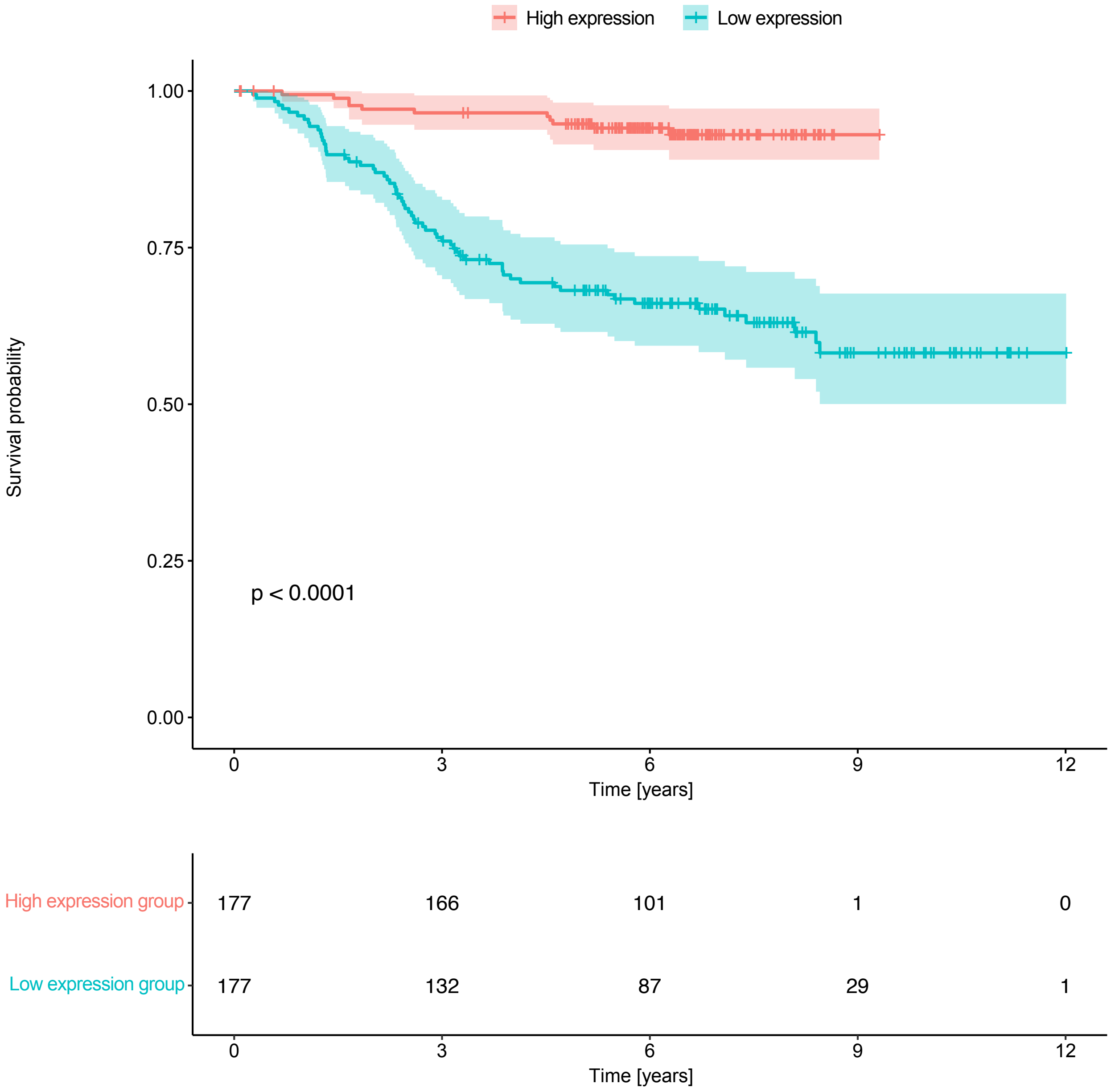

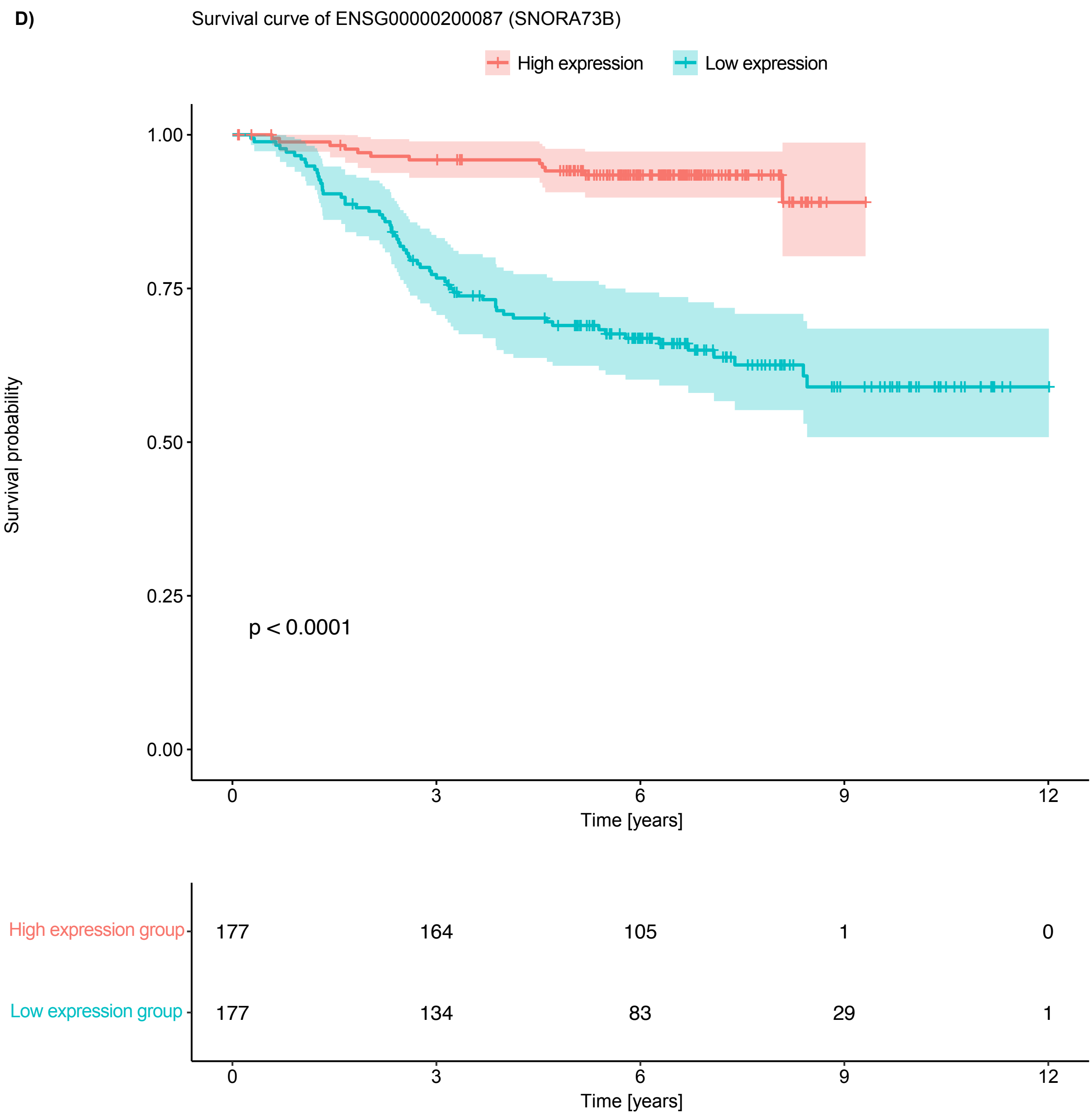
D)
Survival curve of ENSG00000200087 (SNORA73B)
High expression
Low expression
Survival probability
1.00
0.75
0.50
0.25
0.00
p < 0.0001
0
3
6
9
12
Time [years]
High expression group 177 164 105 1 0
Low expression group 177 134 83 29 1
0
3
6
9
12
Time [years]

E)

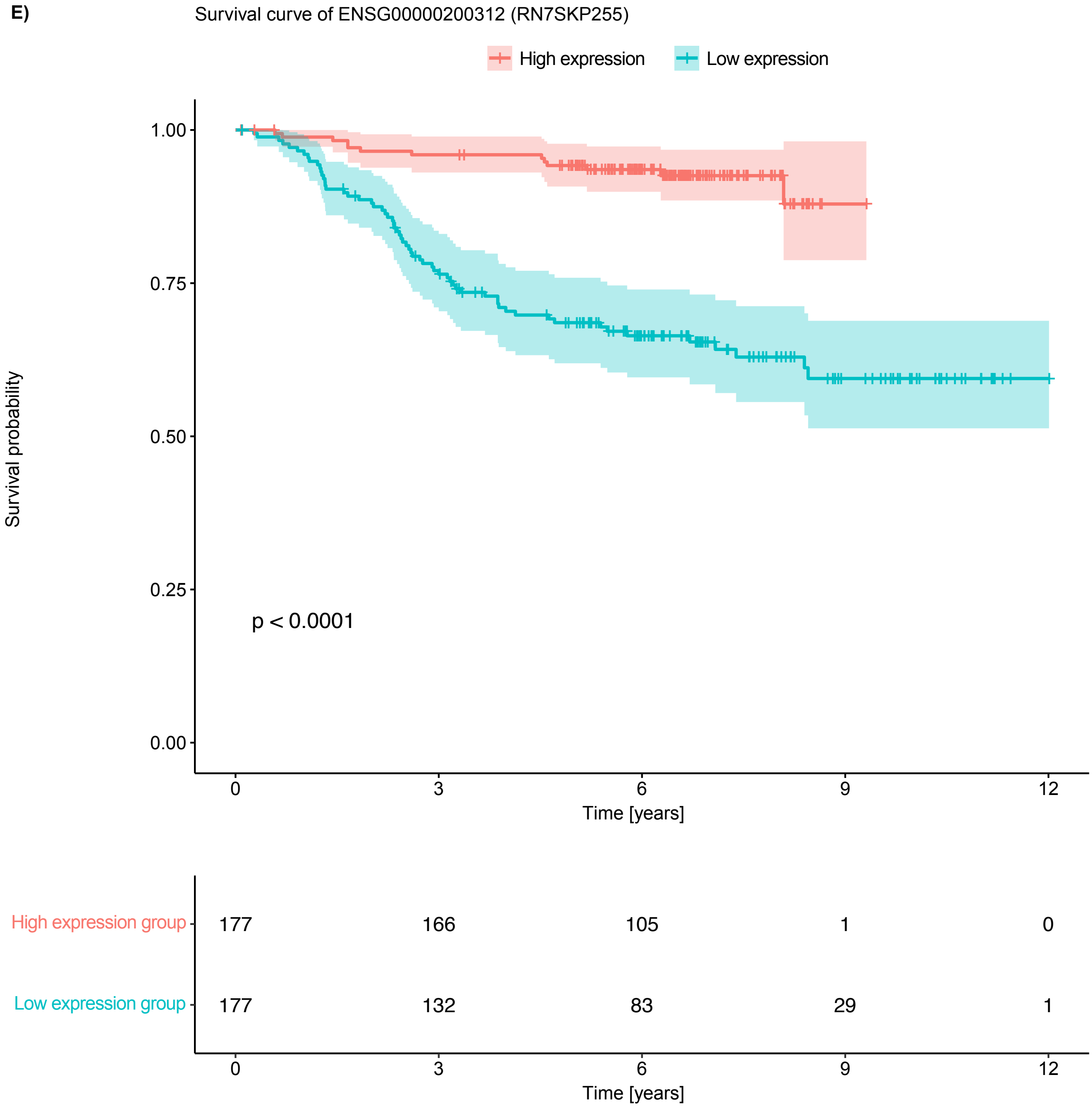

Survival curve of ENSG00000200312 (RN7SKP255)
High expression
Low expression
1.00
0.75
0.50
0.25
0.00
Survival probability
p < 0.0001
0
3
6
9
12
Time [years]
High expression group
Low expression group
177
166
105
1
0
177
132
83
29
1
0
3
6
9
12
Time [years]

F)

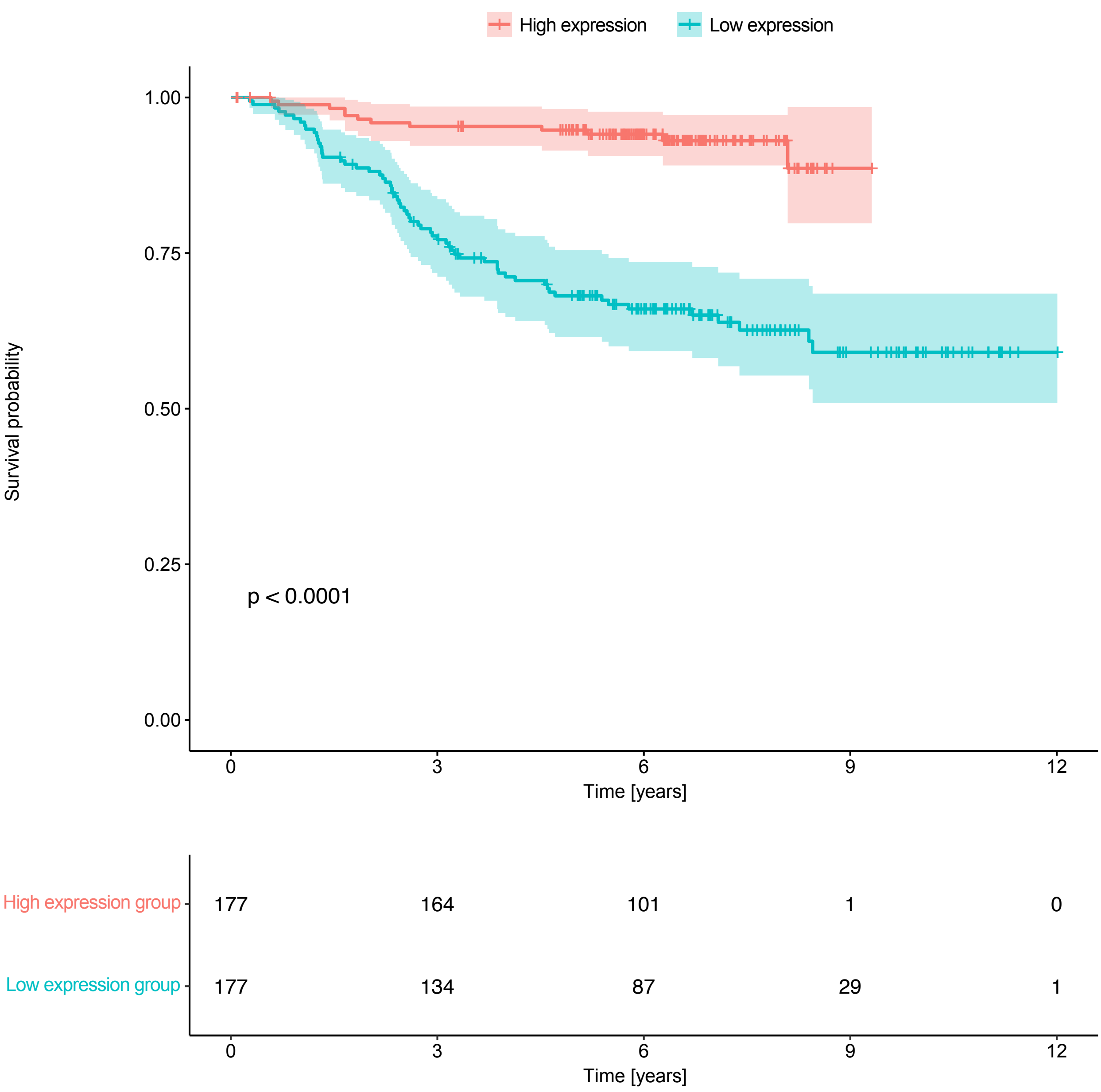
Survival curve of ENSG00000200959 (SNORA74A)
High expression
Low expression
Survival probability
1.00
0.75
0.50
0.25
0.00
p < 0.0001
0
3
6
9
12
Time [years]
High expression group
177
164
101
1
0
Low expression group
177
134
87
29
1
0
3
6
9
12
Time [years]

G) Survival curve of ENSG00000201901 (RN7SKP48)

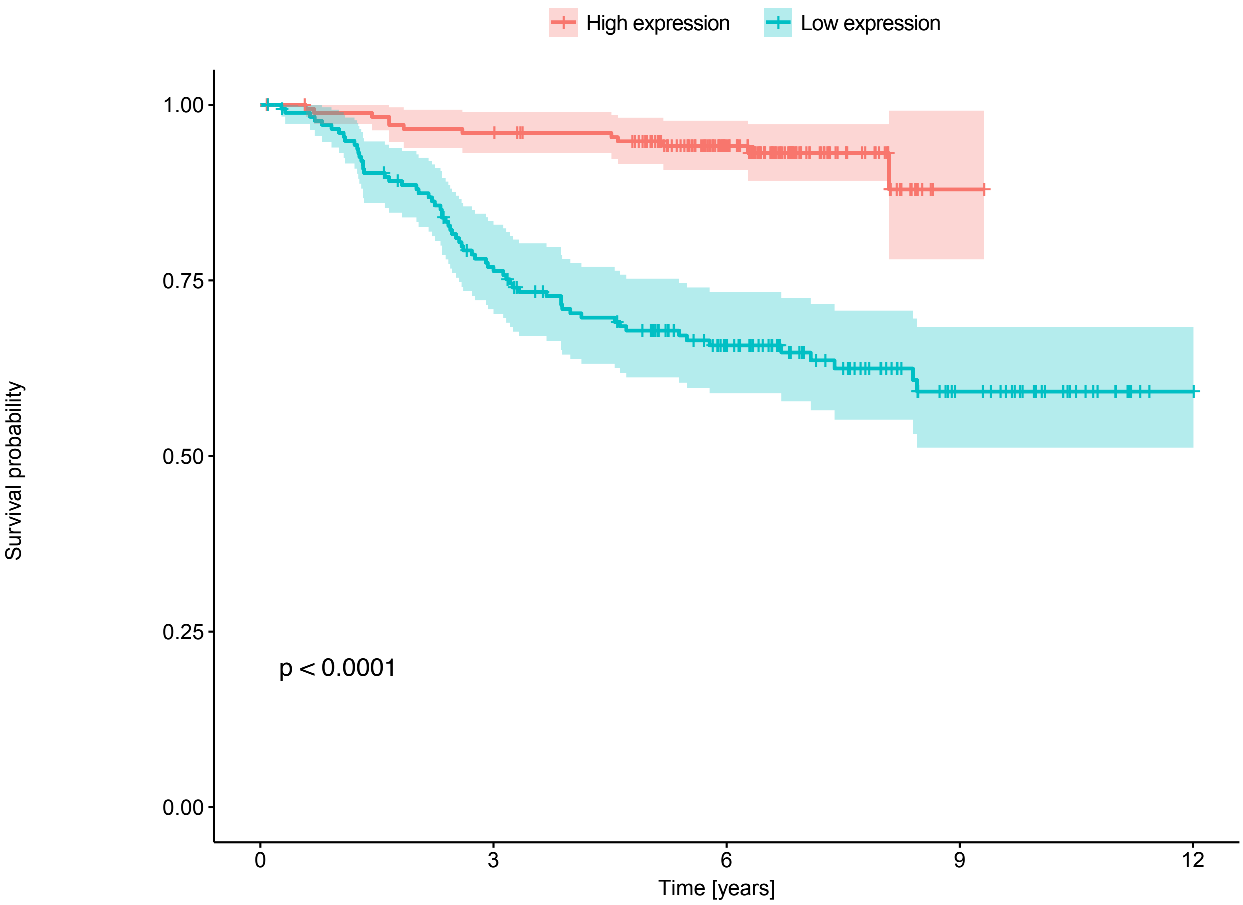

	0	3	6	9	12
High expression group	177	167	105	1	0
Low expression group	177	131	83	29	1

Time [years]

H)

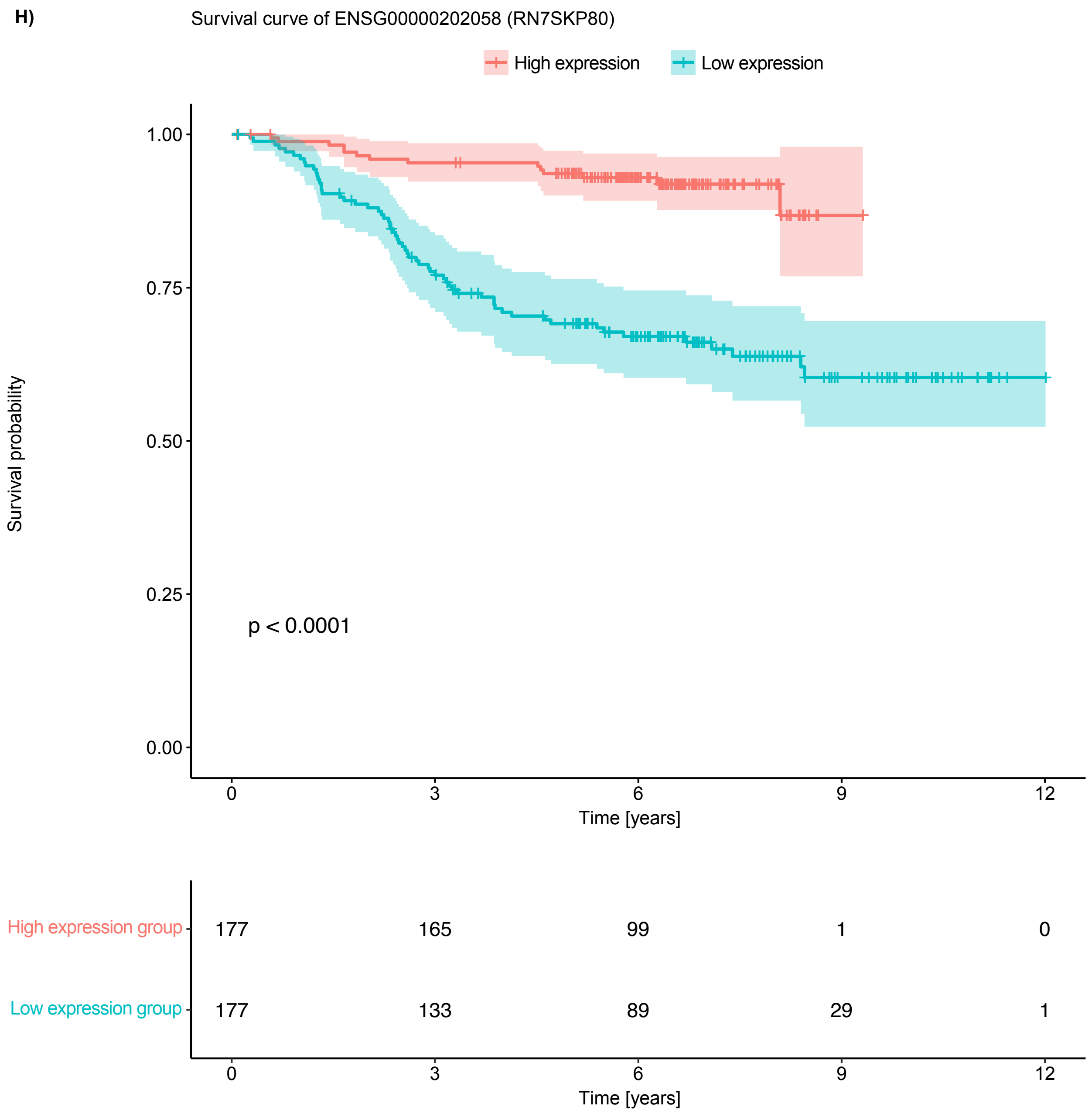
Survival curve of ENSG00000202058 (RN7SKP80)
High expression
Low expression
Survival probability
1.00
0.75
0.50
0.25
0.00
p < 0.0001
0
3
6
9
12
Time [years]
High expression group
177
165
99
1
0
Low expression group
177
133
89
29
1
Time [years]

I)

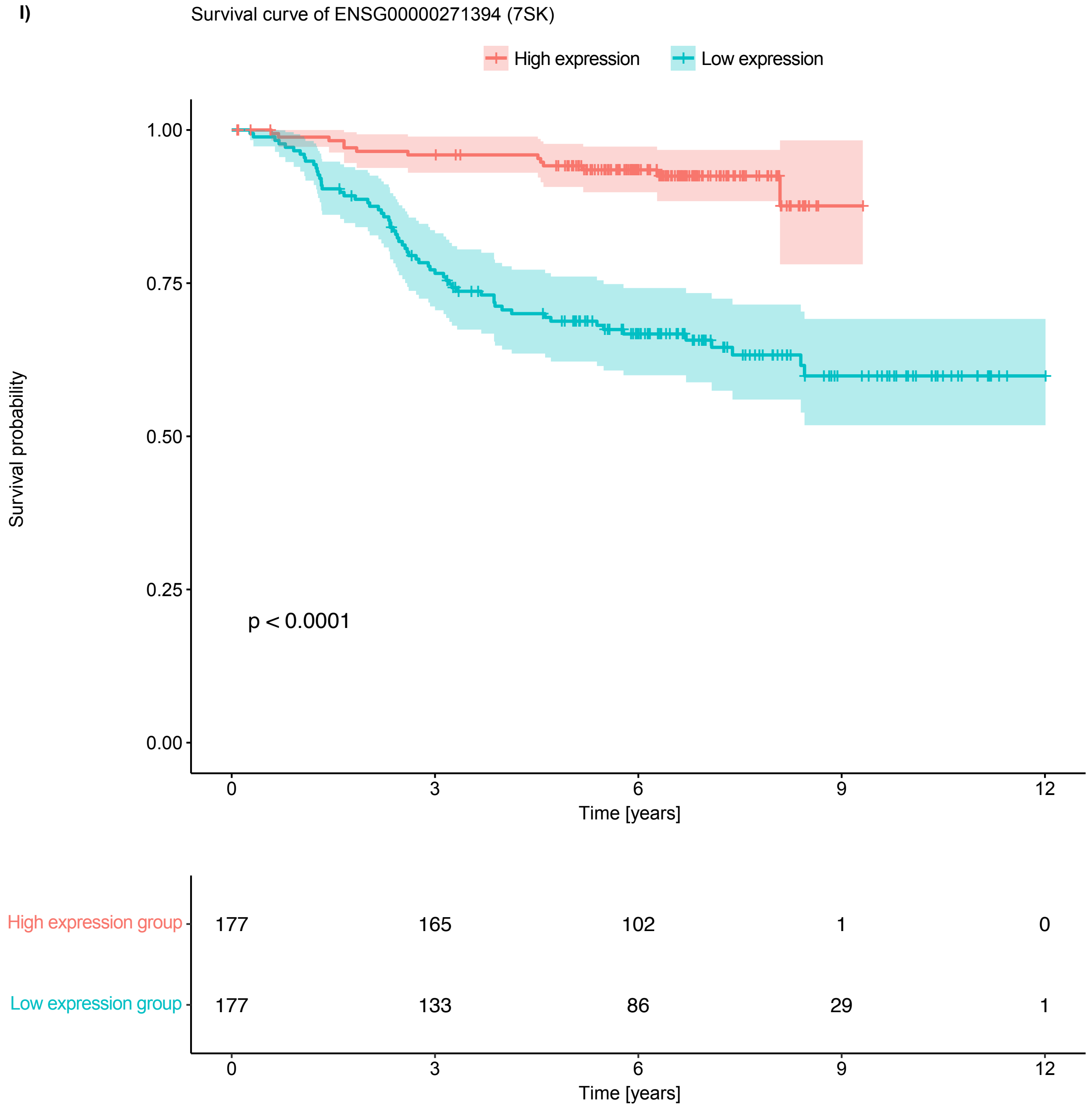

Supplementary Figure S5. Kaplan-Meier survival plots of nine of the discovered subtype-related gene expression markers: **A)** *NDST3*, **B)** RN7SKP185, **C)** RN7SKP291, **D)** SNORA73B, **E)** RN7SKP255, **F)** SNORA74A, **G)** RN7SKP48, **H)** RN7SKP80, and **I)** 7SK**.** Patients were categorized into high (orange) and low (blue) expression groups based on whether their expression values were above or below the median expression level of the corresponding gene. Survival curves were constructed using the discrete expression group as the independent variable, and the significance of the difference in survival between the two groups was assessed using a log-rank test with a *p*-value < 0.05 considered statistically significant.
