# Supplementary material for: Data‐driven discovery of gene expression markers distinguishing pediatric acute lymphoblastic leukemia subtypes
Source: Mol Oncol. 2025 Aug 11;19(12):3548–77. doi: 10.1002/1878-0261.70046 (PMC12688183; doi:10.1002/1878-0261.70046)
Supplement: Supplementary file 6 — Fig. S6. Visualization of unsupervised clustering using method SD:mclust. [file MOL2-19-3548-s006.pdf]

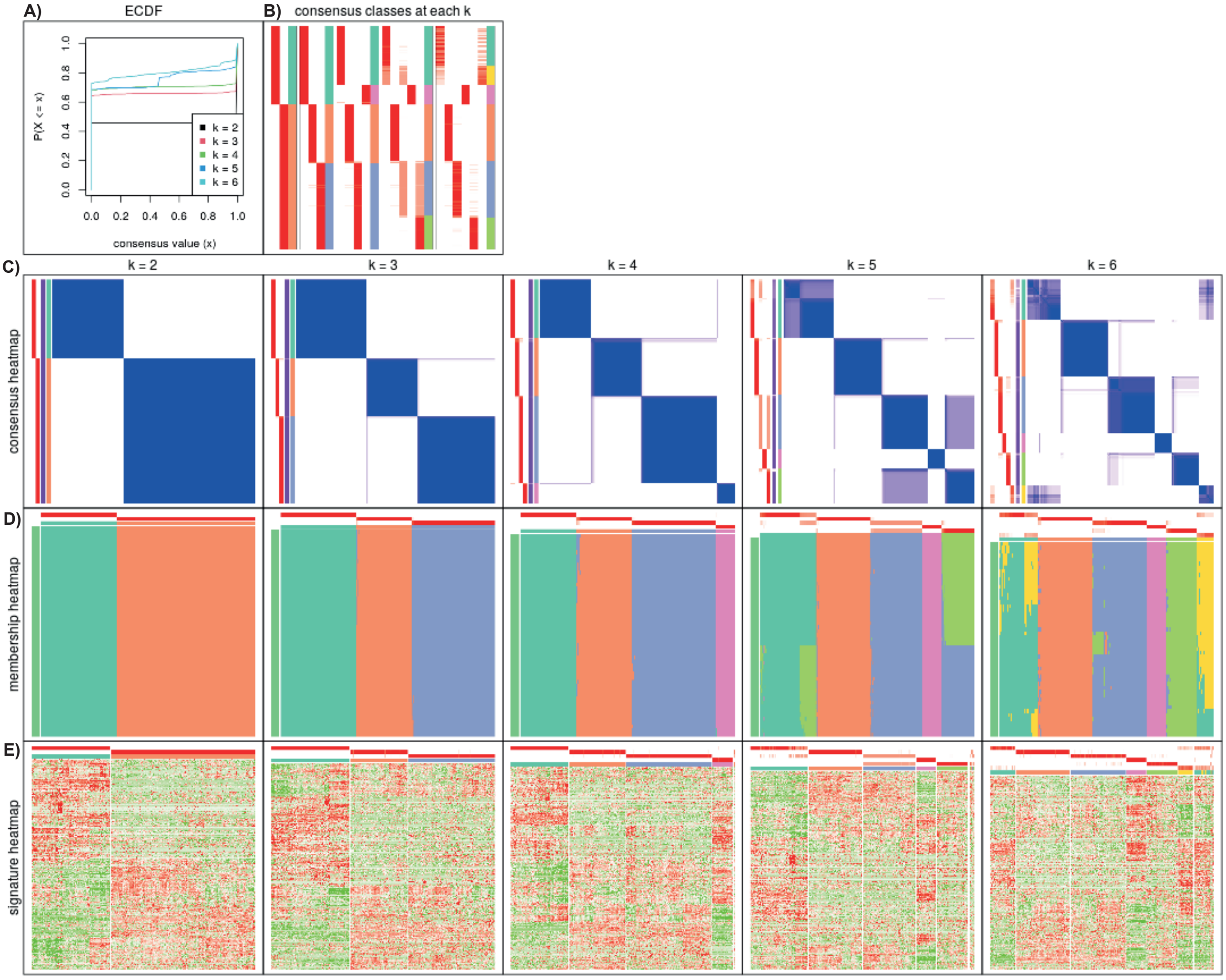

Supplementary Figure S6. Visualization of unsupervised clustering using method standard deviation:model-based clustering (SD:mclust). The unsupervised clustering was performed using the *cola* framework. **A)** Empirical cumulative distribution function curve of the consensus matrix for all values of *k*. **B)** Heatmap of predicted classes showing probability of samples (rows) to belong to *k* number of classes. **C)** Consensus heatmaps showing probability of two samples to be in the same subgroup across all partitions. **D)** Membership heatmaps visualizing the subgroup label that each partition (rows) predicts the samples (columns) to belong to. **E)** Signature heatmaps showing the genes with significant differences in expression between subgroups.
