# Supplementary material for: Data‐driven discovery of gene expression markers distinguishing pediatric acute lymphoblastic leukemia subtypes
Source: Mol Oncol. 2025 Aug 11;19(12):3548–77. doi: 10.1002/1878-0261.70046 (PMC12688183; doi:10.1002/1878-0261.70046)
Supplement: Supplementary file 7 — Fig. S7. Contributions in % of the top 50 genes to principal component 1 and principal component 2 performed on gene expression data of a Danish cohort of pediatric patients with ALL. [file MOL2-19-3548-s012.pdf]

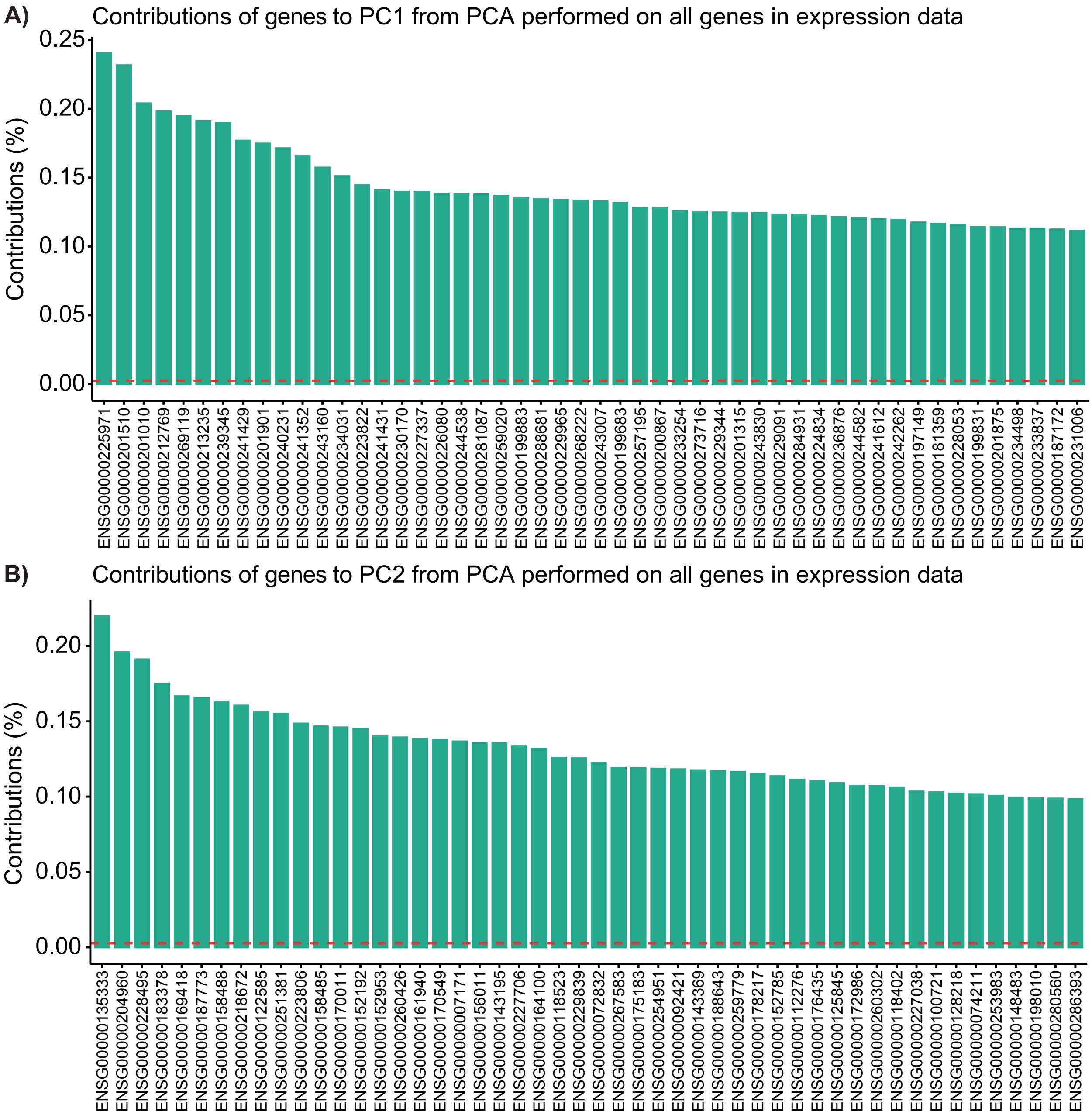

Supplementary Figure S7. Contributions in % of the top 50 genes to principal component 1 (PC1) **A)** and principal component 2 (PC2) **B)**. The principal component analysis (PCA) was performed on gene expression data of a Danish cohort of pediatric patients with acute lymphoblastic leukemia (ALL).
