# Supplementary material for: Data‐driven discovery of gene expression markers distinguishing pediatric acute lymphoblastic leukemia subtypes
Source: Mol Oncol. 2025 Aug 11;19(12):3548–77. doi: 10.1002/1878-0261.70046 (PMC12688183; doi:10.1002/1878-0261.70046)
Supplement: Supplementary file 8 — Fig. S8. Unsupervised hierarchical clustering of expression data of the 14 markers visualized as heatmaps of additional datasets. [file MOL2-19-3548-s007.pdf]

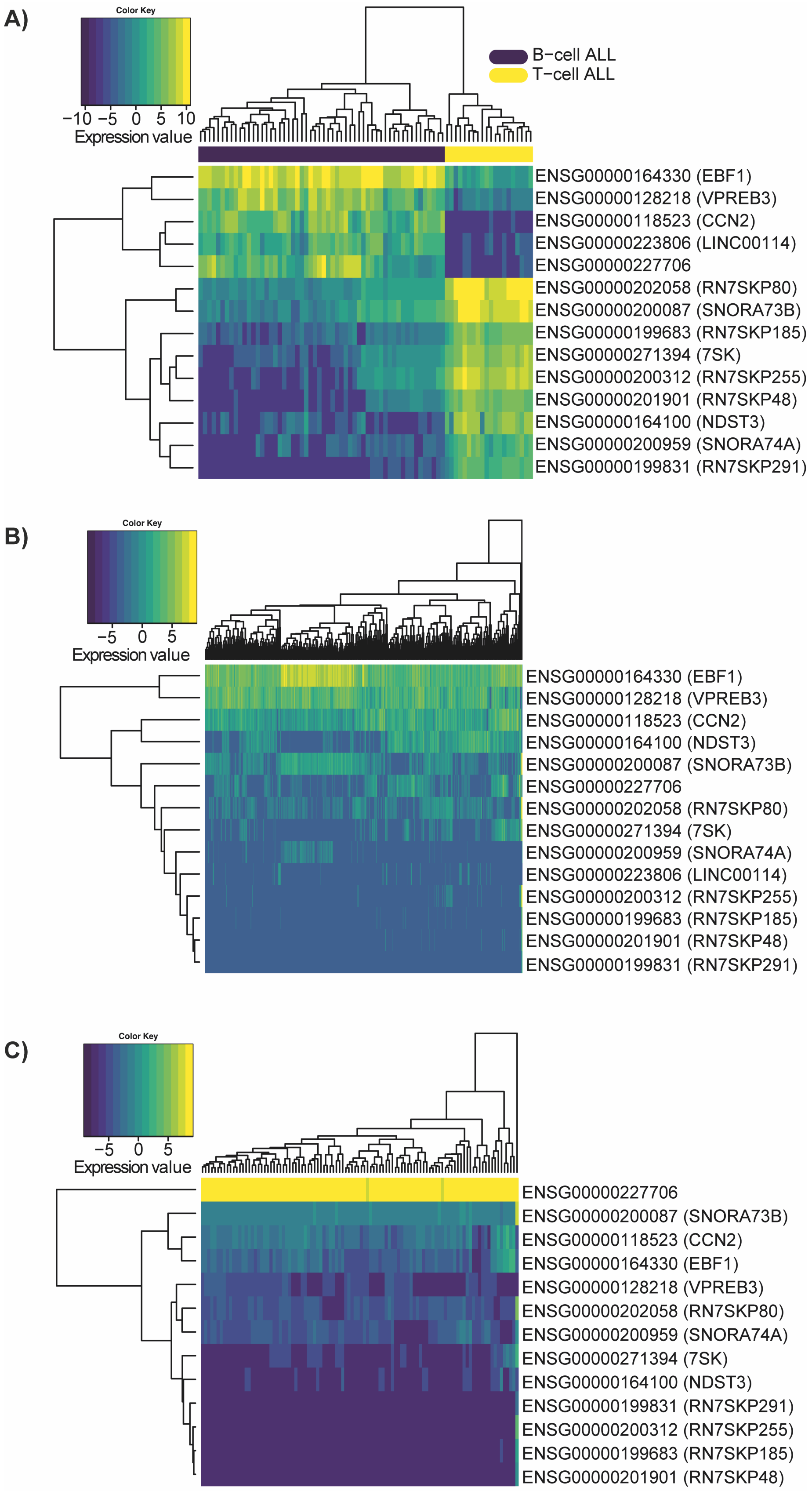
A)
Color Key
−10 −5 0 5 10
Expression value
B−cell ALL
T−cell ALL
ENSG00000164330 (EBF1)
ENSG00000128218 (VPREB3)
ENSG00000118523 (CCN2)
ENSG00000223806 (LINC00114)
ENSG00000227706
ENSG00000202058 (RN7SKP80)
ENSG00000200087 (SNORA73B)
ENSG00000199683 (RN7SKP185)
ENSG00000271394 (7SK)
ENSG00000200312 (RN7SKP255)
ENSG00000201901 (RN7SKP48)
ENSG00000164100 (NDST3)
ENSG00000200959 (SNORA74A)
ENSG00000199831 (RN7SKP291)
B)
Color Key
−5 0 5
Expression value
ENSG00000164330 (EBF1)
ENSG00000128218 (VPREB3)
ENSG00000118523 (CCN2)
ENSG00000164100 (NDST3)
ENSG00000200087 (SNORA73B)
ENSG00000227706
ENSG00000202058 (RN7SKP80)
ENSG00000271394 (7SK)
ENSG00000200959 (SNORA74A)
ENSG00000223806 (LINC00114)
ENSG00000200312 (RN7SKP255)
ENSG00000199683 (RN7SKP185)
ENSG00000201901 (RN7SKP48)
ENSG00000199831 (RN7SKP291)
C)
Color Key
−5 0 5
Expression value
ENSG00000227706
ENSG00000200087 (SNORA73B)
ENSG00000118523 (CCN2)
ENSG00000164330 (EBF1)
ENSG00000128218 (VPREB3)
ENSG00000202058 (RN7SKP80)
ENSG00000200959 (SNORA74A)
ENSG00000271394 (7SK)
ENSG00000164100 (NDST3)
ENSG00000199831 (RN7SKP291)
ENSG00000200312 (RN7SKP255)
ENSG00000199683 (RN7SKP185)
ENSG00000201901 (RN7SKP48)

Supplementary Figure S8. Unsupervised hierarchical clustering of expression data of the 14 markers in different datasets visualized as heatmaps. Values in the heatmaps are voom transformed processed expression data. **A)** Clustering performed on TARGET blood samples. The samples are annotated with subtype (B- and T-cell acute lymphoblastic leukemia (ALL)) labels. **B)** Clustering performed on Genotype-Tissue Expression (GTEx) blood samples. **C)** Clustering performed on GTEx bone marrow samples. Only 13 of the predicted 14 markers are included in **C)** as the marker, LINC00114, was not retained in the expression matrix following data processing.
