# Supplementary material for: Data‐driven discovery of gene expression markers distinguishing pediatric acute lymphoblastic leukemia subtypes
Source: Mol Oncol. 2025 Aug 11;19(12):3548–77. doi: 10.1002/1878-0261.70046 (PMC12688183; doi:10.1002/1878-0261.70046)
Supplement: Supplementary file 10 — Table S2. List of 103 housekeeping differentially expressed genes. [file MOL2-19-3548-s015.pdf]

Supplementary Table S2

Supplementary Table S2. List of 103 housekeeping differentially expressed genes (DEGs). 103 DEGs overlapped between the list of housekeeping genes provided by Eisenberg and Levanon (2013) and our list of consensus DEGs. The average log2 fold change (log2FC) and average False Discovery Rate (FDR) values of the DEGs are shown (average across three differential expression analysis (DEA) methods: limma-voom, edgeR, and DESeq2).			
**ENSEMBL gene ID**	**External gene name**	**Average log2FC [SD]**	**Average FDR [SD]**
ENSG00000117528	*ABCD3*	-2.0558 [0.2284]	7.8268e-10 [1.3556e-09]
ENSG00000100439	*ABHD4*	2.7184 [0.2267]	4.0769e-13 [7.0268e-13]
ENSG00000189046	*ALKBH2*	-1.6685 [0.2218]	1.1818e-03 [1.5969e-03]
ENSG00000197043	*ANXA6*	-2.0312 [0.2928]	6.7560e-08 [8.8843e-08]
ENSG00000132694	*ARHGEF11*	2.0676 [0.2478]	1.9266e-03 [3.2068e-03]
ENSG00000136950	*ARPC5L*	2.1132 [0.4218]	1.5462e-08 [2.6780e-08]
ENSG00000170653	*ATF7*	1.9854 [0.0941]	3.4806e-24 [6.0287e-24]
ENSG00000017260	*ATP2C1*	-2.0125 [0.2526]	1.2952e-06 [2.2433e-06]
ENSG00000185883	*ATP6V0C*	3.1741 [0.7032]	1.5037e-34 [2.6045e-34]
ENSG00000117475	*BLZF1*	-1.7979 [0.1735]	7.6949e-06 [1.3327e-05]

ENSG00000141867	*BRD4*	1.9672 [0.1583]	3.4038e-08 [5.6954e-08]
ENSG00000171067	*C11orf24*	1.9540 [0.3260]	2.1345e-04 [3.6970e-04]
ENSG00000152492	*CCDC50*	4.5889 [0.8558]	3.0237e-27 [5.1813e-27]
ENSG00000111666	*CHPT1*	1.7283 [0.1110]	2.2142e-04 [3.8067e-04]
ENSG00000140848	*CPNE2*	2.7974 [0.3735]	4.5742e-18 [7.92270e-18]
ENSG00000160741	*CRTC2*	2.2752 [0.4505]	7.6463e-07 [1.3244e-06]
ENSG00000141551	*CSNK1D*	1.7483 [0.3589]	1.1075e-02 [1.9182e-02]
ENSG00000177613	*CSTF2T*	-1.3646 [0.0220]	3.2144e-03 [4.3306e-03]
ENSG00000044115	*CTNNA1*	2.2630 [0.0640]	2.3263e-11 [4.0158e-11]
ENSG00000055130	*CUL1*	2.0240 [0.1984]	2.3276e-07 [4.0132e-07]
ENSG00000100243	*CYB5R3*	1.5671 [0.2937]	1.2558e-02 [2.1751e-02]
ENSG00000273749	*CYFIP1*	2.5279 [0.1503]	4.3537e-14 [7.5153e-14]
ENSG00000155016	*CYP2U1*	-3.2764 [0.0745]	3.9247e-10 [6.797767e-10]

ENSG00000164535	*DAGLB*	3.0061 [0.2123]	4.6799e-32 [8.1057e-32]
ENSG00000112679	*DUSP22*	1.3907 [0.0851]	1.8042e-02 [2.2279e-02]
ENSG00000146425	*DYNLT1*	1.5265 [0.0763]	3.3111e-03 [2.8786e-03]
ENSG00000173812	*EIF1*	1.8970 [0.5505]	9.1656e-03 [1.5875e-02]
ENSG00000114784	*EIF1B*	1.9802 [0.2660]	1.4193e-06 [2.4583e-06]
ENSG00000172071	*EIF2AK3*	2.6319 [0.0589]	1.0763e-18 [1.8640e-18]
ENSG00000118985	*ELL2*	2.8740 [0.1208]	1.8080e-13 [1.5438e-13]
ENSG00000063245	*EPN1*	1.9252 [0.2762]	1.4290e-12 [2.4751e-12]
ENSG00000137166	*FOXP4*	2.3874 [0.6924]	4.0462e-04 [7.0081e-04]
ENSG00000136877	*FPGS*	2.0930 [0.6648]	1.5153e-03 [2.6245e-03]
ENSG00000142252	*GEMIN7*	2.0477 [0.2924]	4.1136e-09 [7.1250e-09]
ENSG00000113552	*GNPDA1*	-2.6470 [0.0455]	3.9711e-09 [6.8768e-09]
ENSG00000114745	*GORASP1*	1.5397 [0.2276]	2.3025e-03 [3.9881e-03]

ENSG00000013583	*HEBP1*	2.1593 [0.0889]	2.5504e-07 [3.5537e-07]
ENSG00000131724	*IL13RA1*	2.1791 [0.2429]	7.8104e-04 [1.2863e-03]
ENSG00000205730	*ITPRIPL2*	4.0302 [0.0750]	2.6791e-20 [4.6404e-20]
ENSG00000109787	*KLF3*	2.1830 [0.2595]	2.0987e-05 [3.5030e-05]
ENSG00000119138	*KLF9*	2.6559 [0.1379]	5.7215e-10 [4.0431e-10]
ENSG00000135686	*KLHL36*	1.9373 [0.0346]	1.5489e-10 [1.7241e-10]
ENSG00000163155	*LYSMD1*	-1.4843 [0.0323]	7.4410e-03 [1.0130e-02]
ENSG00000068305	*MEF2A*	1.4044 [0.0526]	6.4894e-03 [1.0999e-02]
ENSG00000100139	*MICALL1*	1.9195 [0.5175]	6.1831e-03 [1.0709e-02]
ENSG00000175806	*MSRA*	3.7525 [0.6172]	1.1157e-20 [1.7109e-20]
ENSG00000170873	*MTSS1*	1.6227 [0.0843]	1.0680e-02 [8.7850e-03]
ENSG00000188554	*NBR1*	-1.6572 [0.0464]	3.3377e-08 [5.21981e-08]
ENSG00000070614	*NDST1*	2.5957 [0.0510]	3.7965e-10 [4.6511e-10]

ENSG00000141458	*NPC1*	1.9508 [0.2024]	9.5397e-08 [1.6523e-07]
ENSG00000119655	*NPC2*	2.4175 [0.3626]	3.2154e-07 [5.5692e-07]
ENSG00000103148	*NPRL3*	1.6068 [0.1496]	2.1433e-07 [3.7100e-07]
ENSG00000124588	*NQO2*	-1.7752 [0.2779	1.2586e-05 [1.9585e-05]
ENSG00000151623	*NR3C2*	2.4396 [0.5887]	7.7398e-03 [1.3406e-02]
ENSG00000157045	*NTAN1*	1.6784 [0.1135]	9.2072e-08 [1.5895e-07]
ENSG00000183828	*NUDT14*	3.1752 [0.1034]	3.1144e-18 [5.3942e-18]
ENSG00000217930	*PAM16*	1.6526 [0.2599]	1.6941e-04 [2.9343e-04]
ENSG00000163110	*PDLIM5*	-2.2709 [0.1124]	3.9527e-07 [6.8447e-07]
ENSG00000152684	*PELO*	-2.8975 [0.2320]	1.2503e-16 [1.1482e-16]
ENSG00000166821	*PEX11A*	-3.0641 [0.3066]	3.7929e-13 [6.5656e-13]
ENSG00000139197	*PEX5*	-2.6268 [0.3444]	4.0208e-17 [6.9643e-17]
ENSG00000130517	*PGPEP1*	-2.1281 [0.4174]	2.4834e-07 [2.6502e-07]

ENSG00000272391	*POM121C*	1.6201 [0.0851]	1.7061e-07 [2.9195e-07]
ENSG00000112033	*PPARD*	2.4167 [0.3865]	1.2427e-11 [2.1524e-11]
ENSG00000143801	*PSEN2*	2.3613 [0.2920]	7.8223e-04 [1.3541e-03]
ENSG00000130348	*QRSL1*	1.8480 [0.1013]	2.8844e-09 [3.9329e-09]
ENSG00000137040	*RANBP6*	-1.5199 [0.1505]	2.2870e-03 [3.9608e-03]
ENSG00000107263	*RAPGEF1*	1.7829 [0.2424]	1.7698e-05 [3.0654e-05]
ENSG00000183808	*RBM12B*	-1.4516 [0.0764]	2.4313e-03 [4.1415e-03]
ENSG00000159200	*RCAN1*	2.3074 [0.1182]	2.7208e-06 [4.1817e-06]
ENSG00000129625	*REEP5*	1.7002 [0.1148]	1.6964e-05 [2.9292e-05]
ENSG00000173039	*RELA*	1.9231 [0.5209]	3.5427e-04 [6.1361e-04]
ENSG00000143878	*RHOB*	3.3279 [0.3322]	5.6136e-12 [9.7224e-12]
ENSG00000132669	*RIN2*	3.3818 [0.0562]	6.1996e-20 [6.8905e-20]
ENSG00000151748	*SAV1*	2.1427 [0.4001]	4.1695e-07 [7.1196e-07]

ENSG00000138760	*SCARB2*	1.8510 [0.3696]	1.3454e-03 [2.2869e-03]
ENSG00000124570	*SERPINB6*	2.5993 [0.5928]	4.5298e-05 [6.7008e-05]
ENSG00000102100	*SLC35A2*	1.9292 [0.2430]	1.4498e-06 [2.5111e-06]
ENSG00000175782	*SLC35E3*	2.6622 [0.0783]	4.7145e-26 [8.1658e-26]
ENSG00000205302	*SNX2*	2.5882 [0.2717]	3.7825e-38 [3.4408e-38]
ENSG00000109762	*SNX25*	2.2784 [0.1487]	2.6136e-05 [3.6068e-05]
ENSG00000130340	*SNX9*	3.0137 [0.0850]	5.2106e-13 [7.8938e-13]
ENSG00000123178	*SPRYD7*	2.0087 [0.0868]	6.9119e-08 [5.9435e-08]
ENSG00000100104	*SRRD*	1.3270 [0.0526]	4.6980e-04 [4.2679e-04]
ENSG00000040341	*STAU2*	-2.1003 [0.1069]	2.9640e-09 [5.1337e-09]
ENSG00000100242	*SUN2*	1.5278 [0.1522]	2.1608e-03 [3.7423e-03]
ENSG00000162298	*SYVN1*	3.0607 [0.3542]	3.1243e-45 [5.4115e-45]
ENSG00000169762	*TAPT1*	1.5817 [0.0339]	1.6043e-03 [2.4792e-03]

ENSG00000132604	*TERF2*	2.0445 [0.2279]	6.1102e-11 [1.0583e-10]
ENSG00000127666	*TICAM1*	2.3204 [0.4467]	4.8051e-08 [8.3228e-08]
ENSG00000196781	*TLE1*	3.1004 [0.0917]	7.3106e-14 [1.2418e-13]
ENSG00000137842	*TMEM62*	2.0251 [0.3619]	3.8211e-06 [6.5942e-06]
ENSG00000180694	*TMEM64*	2.4424 [0.8092]	5.6949e-04 [9.2631e-04]
ENSG00000125827	*TMX4*	1.6811 [0.1878]	8.2547e-05 [1.4190e-04]
ENSG00000243725	*TTC4*	-1.4174 [0.1327]	6.5920e-03 [6.6144e-03]
ENSG00000153443	*UBALD1*	2.5131 [0.5384]	2.8853e-16 [4.9974e-16]
ENSG00000198833	*UBE2J1*	1.5392 [0.1807]	4.2615e-03 [7.2222e-03]
ENSG00000115652	*UXS1*	1.3878 [0.1048]	4.5820e-03 [7.8179e-03]
ENSG00000140006	*WDR89*	-1.7109 [0.0459]	5.5711e-09 [6.4233e-09]
ENSG00000175155	*YPEL2*	2.0539 [0.1143]	3.7284e-10 [6.3663e-10]
ENSG00000147905	*ZCCHC7*	2.5712 [0.1514]	1.2016e-40 [2.0804e-40]

ENSG00000178764	*ZHX2*	2.1086 [0.2899]	1.1163e-10 [1.9334e-10]
ENSG00000179195	*ZNF664*	-1.4443 [0.1614]	2.7303e-03 [3.8936e-03]
Abbreviations: DEGs, differentially expressed genes; log2FC, log2 fold change; FDR, false discovery rate; DEA, differential expression analysis; SD, standard deviation.			
