# Supplementary material for: Data‐driven discovery of gene expression markers distinguishing pediatric acute lymphoblastic leukemia subtypes
Source: Mol Oncol. 2025 Aug 11;19(12):3548–77. doi: 10.1002/1878-0261.70046 (PMC12688183; doi:10.1002/1878-0261.70046)
Supplement: Supplementary file 11 — Table S3. Consensus set of selected ENSEMBL gene IDs from elastic net binomial logistic regression. [file MOL2-19-3548-s010.pdf]

Supplementary Table S3

**Supplementary Table S3. Consensus set of selected ENSEMBL gene IDs from elastic net binomial logistic regression together with their external gene name and average coefficient sorted in descending order by absolute average coefficient. We created the consensus set of ENSEMBL gene IDs based on overlap of selected ENSEMBL gene IDs from 10 elastic net logistic regression runs. The consensus genes are compared with results of the consensus differential expression analysis (DEA).**				
**ENSEMBL gene ID**	**External gene name [biotype]**	**Average elastic net coefficient [SD]**	**Average log2FC [SD]**	**Average FDR [SD]**
ENSG00000243062	Novel gene [lncRNA]	0.0179 [0.002]	-5.4104 [0.5948]	7.0414e-53 [1.1355e-52]
ENSG00000199831	RN7SKP291 [misc RNA]	0.0137 [0.0004]	-9.3511 [0.2172]	5.5305e-87 [9.5791e-87]
ENSG00000202058	RN7SKP80 [misc RNA]	0.0116 [0.0003]	-8.7568 [0.7806]	3.3216e-80 [5.7532e-80]
ENSG00000228997	Novel gene [processed pseudogene]	0.0087 [0.0018]	-5.6471 [0.1568]	3.7650e-42 [6.5211e-42]
ENSG00000275155	Novel gene [lncRNA]	-0.0080 [0.0013]	6.4042 [0.3272]	3.8950e-79 [6.7463e-79]
ENSG00000202357	Y_RNA [misc RNA]	0.0079 [0.0009]	-7.9692 [0.7869]	3.6584e-55 [6.3366e-55]
ENSG00000164100	NDST3 [protein coding]	0.0076 [0.0005]	-11.6426 [0.3028]	2.1479e-77 [3.7186e-77]
ENSG00000199683	RN7SKP185 [misc RNA]	0.0073 [0.0007]	-7.7758 [0.6542]	1.1201e-70 [1.5208e-70]
ENSG00000279204	Novel gene [TEC]	-0.0067 [0.0007]	7.2987 [0.1192]	6.1784e-92 [1.0701e-91]
ENSG00000095585	BLNK [protein coding]	-0.0063 [0.0009]	7.0636 [0.4600]	7.1782e-93 [1.2433e-92]
ENSG00000223806	LINC00114 [lncRNA]	-0.0062 [0.0009]	8.7047 [0.2038]	2.9924e-87 [5.1830e-87]
ENSG00000225206	MIR137HG [lncRNA]	0.0055 [0.0006]	-7.5747 [0.5870]	3.1520e-52 [5.4594e-52]
ENSG00000118523	CCN2 [protein coding]	-0.0054 [0.0010]	9.3982 [0.2464]	2.3999e-81 [4.1568e-81]

ENSG00000238741	SCARNA7 [scaRNA]	0.0054 [0.0011]	-6.7528 [0.0907]	1.8558e-48 [3.2144e-48]
ENSG00000200959	SNORA74A [snoRNA]	0.0052 [0.0006]	-8.6670 [0.3967]	1.3186e-58 [2.2837e-58]
ENSG00000200087	SNORA73B [snoRNA]	0.0050 [0.0005]	-7.3992 [0.9972]	2.1027e-44 [3.6421e-44]
ENSG00000263413	MIR4538 [miRNA]	-0.0049 [0.0024]	6.6022 [1.6395]	1.8674e-66 [3.2344e-66]
ENSG00000105694	ELOCP28 [processed pseudogene]	-0.0047 [0.0022]	6.3175 [0.1198]	6.6892e-59 [1.1586e-58]
ENSG00000201901	RN7SKP48 [misc RNA]	0.0042 [0.0005]	-7.6915 [1.0032]	2.3876e-47 [4.1355e-47]
ENSG00000177455	CD19 [protein coding]	-0.0042 [0.0009]	7.4110 [0.1468]	2.3812e-118 [4.1243e-118]
ENSG00000212232	SNORD17 [snoRNA]	0.0041 [0.0009]	-7.0919 [0.6947]	5.0064e-42 [8.6714e-42]
ENSG00000164330	EBF1 [protein coding]	-0.0038 [0.0008]	8.1850 [0.7341]	9.4079e-103 [1.6295e-102]
ENSG00000200795	RNU4-1 [snRNA]	0.0035 [0.0007]	-6.6895 [0.4326]	8.4889e-45 [1.4703e-44]
ENSG00000196092	PAX5 [protein coding]	-0.0033 [0.0007]	7.5755 [0.2704]	3.9762e-107 [6.8870e-107]
ENSG00000200312	RN7SKP255 [misc RNA]	0.0029 [0.0006]	-8.1543 [1.5475]	8.8446e-38 [1.5319e-37]
ENSG00000145642	SHISAL2B [protein coding]	0.0029 [0.0014]	-5.3578 [0.5424]	2.1131e-51 [3.6599e-51]
ENSG00000183918	SH2D1A [protein coding]	0.0027 [0.0016]	-5.7853 [0.0451]	4.6729e-49 [8.0937e-49]
ENSG00000227706	Novel gene [lncRNA]	-0.0026 [0.0009]	11.6906 [1.7298]	1.0997e-90 [1.9047e-90]
ENSG00000200314	Y_RNA [misc RNA]	0.0017 [0.0013]	-8.3174 [0.8292]	1.5658e-65 [2.7120e-65]
ENSG00000271394	7SK [misc RNA]	0.0014 [0.0007]	-7.4783 [0.7991]	3.7501e-47 [6.4834e-47]
ENSG00000128218	VPREB3 [protein coding]	-0.0011 [0.0007]	9.2479 [0.4986]	1.0546e-106 [1.8266e-106]

Abbreviations: DEA, differential expression analysis; SD, standard deviation; log2FC, log2 fold change; FDR, false discovery rate.
