# Supplementary material for: Data‐driven discovery of gene expression markers distinguishing pediatric acute lymphoblastic leukemia subtypes
Source: Mol Oncol. 2025 Aug 11;19(12):3548–77. doi: 10.1002/1878-0261.70046 (PMC12688183; doi:10.1002/1878-0261.70046)
Supplement: Supplementary file 12 — Table S4. Top 40 ENSEMBL gene IDs with highest contribution of explained variance between acute lymphoblastic leukemia (ALL) samples along the first principal component. [file MOL2-19-3548-s001.pdf]

Supplementary Table S4

Supplementary Table S4. Top 40 ENSEMBL gene IDs with highest contribution of explained variance between acute lymphoblastic leukemia (ALL) samples along the first principal component. ENSEMBL gene ID, external gene, biotype, and contribution of these 40 ENSEMBL gene IDs sorted by contribution in percentage. The ENSEMBL gene IDs are compared with results of the consensus differential expression analysis (DEA).				
**ENSEMBL gene ID**	**External gene name [biotype]**	**Contribution in %**	**Average log2FC [SD]**	**Average FDR [SD]**
ENSG00000164100	NDST3 [protein coding]	0.171	-11.6426 [0.3028]	2.1470e-77 [3.7186e-77]
ENSG00000158488	CD1E [protein coding]	0.1444	-11.4757 [1.0199]	7.1443e-16 [1.2374e-15]
ENSG00000158485	CD1B [protein coding]	0.1349	-10.5611 [0.6251]	5.8946e-18 [1.0210e-17]
ENSG00000200312	RN7SKP255 [miscRNA]	0.1252	-8.1543 [1.5475]	8.8446e-38 [1.5319e-37]
ENSG00000200488	RN7SKP203 [miscRNA]	0.1173	-7.9260 [1.4508]	1.6326e-36 [2.8277e-36]
ENSG00000227706	Novel gene [lncRNA]	0.1136	11.6906 [1.7298]	1.0997e-90 [1.9047e-90]
ENSG00000202058	RN7SKP80 [miscRNA]	0.1099	-8.7568 [0.7806]	3.3216e-80 [5.7532e-80]
ENSG00000199831	RN7SKP291 [miscRNA]	0.107	-9.3511 [0.2172]	5.5305e-87 [9.5791e-87]
ENSG00000204960	BLACE [lncRNA]	0.1023	10.4528 [0.9408]	1.2670e-59 [2.1946e-59]
ENSG00000118523	CCN2 [protein coding]	0.1011	9.3982 [0.2464]	2.3999e-81 [4.1568e-81]
ENSG00000118402	ELOVL4 [protein coding]	0.0999	-9.0693 [0.2217]	3.1359e-19 [5.4316e-19]
ENSG00000172986	GXYLT2 [protein coding]	0.0977	-7.4779 [1.2998]	2.1511e-30 [2.4827e-30]
ENSG00000138650	PCDH10 [protein coding]	0.0973	-8.0614 [0.9515]	2.5417e-16 [4.3634e-16]
ENSG00000196581	AJAP1 [protein coding]	0.0961	-5.7711 [2.4977]	7.3267e-09 [1.2690e-08]
ENSG00000201	RN7SKP48	0.0952	-7.6915 [1.0032]	2.3876e-47

901	[miscRNA]			[4.1355e-47]
ENSG00000228495	LINC01013 [lncRNA]	0.0948	8.6272 [0.2294]	4.3675e-89 [7.5647e-89]
ENSG00000164330	EBF1 [protein coding]	0.0941	8.1850 [0.7341]	9.4078e-103 [1.6295e-102]
ENSG00000236656	Novel gene [lncRNA]	0.094	-10.4154 [2.1106]	3.6629e-16 [6.3444e-16]
ENSG00000200959	SNORA74A [snoRNA]	0.0938	-8.6670 [0.3967]	1.3186e-58 [2.2837e-58]
ENSG00000223806	LINC00114 [lncRNA]	0.0927	8.7047 [0.2038]	2.9924e-87 [5.1830e-87]
ENSG00000271394	7SK [miscRNA]	0.0914	-7.4783 [0.7991]	3.7501e-47 [6.4834e-47]
ENSG00000100721	TCL1A [protein coding]	0.0896	8.8481 [0.1984]	5.3461e-85 [9.2597e-85]
ENSG00000199683	RN7SKP185 [miscRNA]	0.0864	-7.7758 [0.6542]	1.1201e-70 [1.5208e-70]
ENSG00000188643	S100A16 [protein coding]	0.0855	10.5736 [1.9572]	1.3122e-59 [2.2729e-59]
ENSG00000128218	VPREB3 [protein coding]	0.0841	9.2479 [0.4986]	1.0546e-106 [1.8266e-106]
ENSG00000200087	SNORA73B [snoRNA]	0.0834	-7.3992 [0.9972]	2.1027e-44 [3.6420e-44]
ENSG00000136531	SCN2A [protein coding]	0.0802	-8.1226 [0.0530]	2.0875e-11 [3.6157e-11]
ENSG00000267206	LCN6 [protein coding]	0.0797	7.7130 [0.5306]	1.4949e-40 [2.5893e-40]
ENSG00000263667	Novel gene [lncRNA]	0.0794	7.9739 [0.6103]	4.2172e-54 [7.3005e-54]
ENSG00000125845	BMP2 [protein coding]	0.0792	8.1838 [0.1371]	3.4885e-54 [6.0421e-54]
ENSG00000254535	PABPC4L [protein coding]	0.079	-7.1982 [0.7380]	1.3277e-08 [2.2996e-08]
ENSG00000150722	PPP1R1C [protein coding]	0.079	-7.7929 [0.1759]	5.8328e-23 [1.0103e-22]
ENSG00000149256	TENM4 [protein coding]	0.0783	8.6083 [0.1512]	7.0158e-50 [1.2152e-49]

ENSG00000253883	IGHV3-19 [pseudogene]	0.0777	-8.7809 [0.8666]	7.6242e-46 [1.3205e-45]
ENSG00000187621	TCL6 [lncRNA]	0.0772	8.4657 [0.0559]	3.4849e-71 [6.0361e-71]
ENSG00000276778	LINC02227 [lncRNA]	0.0765	7.0439 [1.3625]	1.2184e-38 [2.1025e-38]
ENSG00000161544	CYGB [protein coding]	0.0763	7.9310 [0.0436]	2.4930e-70 [3.8479e-70]
ENSG00000170558	CDH2 [protein coding]	0.076	-5.1614 [2.2585]	1.1728e-08 [1.4038e-08]
ENSG00000128918	ALDH1A2 [protein coding]	0.0757	-8.6717 [1.1190]	1.7997e-07 [3.1172e-07]
ENSG00000251381	LINC00958 [lncRNA]	0.0757	8.2410 [0.3514]	6.3176e-57 [1.0942e-56]

Abbreviations: ALL, acute lymphoblastic leukemia; DEA, differential expression analysis; log2FC, log2 fold change; SD, standard deviation; FDR, false discovery rate.
