# Supplementary material for: Data‐driven discovery of gene expression markers distinguishing pediatric acute lymphoblastic leukemia subtypes
Source: Mol Oncol. 2025 Aug 11;19(12):3548–77. doi: 10.1002/1878-0261.70046 (PMC12688183; doi:10.1002/1878-0261.70046)
Supplement: Supplementary file 15 — Table S7. Results of consensus differential expression analysis of typical surface markers of B and T cells. [file MOL2-19-3548-s018.pdf]

Supplementary Table S7

**Supplementary Table S7. Results of consensus differential expression analysis (DEA) of typical surface markers of B and T cells. The mean log2 fold change (log2FC) and mean False Discovery Rate (FDR) values of the markers are shown (mean across three DEA methods: limma-voom, edgeR, and DESeq2). The rankings of the markers among up- and downregulated consensus differentially expressed genes (DEGs) are shown. CD38 was not found to be significantly differentially expressed in any of the three DEA methods.**				
**B-cell marker**				
**Gene**	**ENSEMBL ID**	**Mean log2FC [sd]**	**Mean FDR [sd]**	**Rank among upregulated consensus DEGs**
CD19	ENSG00000177455	7.41 [0.145]	2.38e-118 [4.12e-118]	33
CD22	ENSG00000012124	6.51 [0.069]	1.12e-92 [1.95e-92]	57
CD24	ENSG00000272398	6.28 [0.125]	4.18e-56 [7.25e-56]	71
CD34	ENSG00000174059	2.43 [0.312]	0.003 [0.003]	999
CD38	ENSG00000004468	NA	NA	NA
CD79a	ENSG00000105369	4.64 [0.762]	3.04e-41 [5.27e-41]	210
**T-cell marker**				
**Gene**	**ENSEMBL ID**	**Mean log2FC [sd]**	**Mean FDR [sd]**	**Rank among downregulated consensus DEGs**
CD1a	ENSG00000158477	-6.01 [0.210]	2.86e-08 [4.95e-08]	89
CD2	ENSG00000116824	-3.47 [0.195]	6.59e-09 [1.14e-08]	624
CD4	ENSG00000010610	-2.06 [0.351]	0.005 [0.003]	1634
CD5	ENSG00000110448	-2.33 [0.566]	1.05e-06 [1.75e-06]	1393
CD7	ENSG00000173762	-3.45 [0.821]	3.92e-13 [6.79e-13]	639
Abbreviations: DEGs, differentially expressed genes; log2FC, log2 fold change; FDR, false discovery rate; DEA, differential expression analysis; SD, standard deviation.				
