# Supplementary material for: Data‐driven discovery of gene expression markers distinguishing pediatric acute lymphoblastic leukemia subtypes
Source: Mol Oncol. 2025 Aug 11;19(12):3548–77. doi: 10.1002/1878-0261.70046 (PMC12688183; doi:10.1002/1878-0261.70046)
Supplement: Supplementary file 17 — Text S2. Details of pipeline settings of RNA sequencing pipeline. [file MOL2-19-3548-s011.pdf]

Supplementary Text S2

Pipeline settings of RNA sequencing pipeline

The pipeline is using Snakemake workflow manager [1] ensuring high level of reproducibility, portability, and parallelization. The pipeline is available on GitHub: https://github.com/ELELAB/RNA_DE_pipeline.

Trimming

Reads were trimmed using Cutadapt [2]. Specifically, adapters and reads shorter than 35 bp were removed.
Params:
`"--minimum-length 35 -a AGAGCACACGTCTGAACTCCAGTCAC -g AGATCGGAAGAGCACACGT -A AGAGCACACGTCTGAACTCCAGTCAC -G AGATCGGAAGAGCACACGT"`

Read mapping

As a first step, reference genome hg38 release 104 was indexed using STAR [3] using transcript annotations GENCODE v38.
Params:
`"--sjdbGTFfile resources/genome.gtf --sjdbOverhang 149"`

Next, pair-end reads were aligned using STAR aligner onto the reference genome. Multimapping reads were retained in order to keep information about possible contamination e.g. by rRNA.
Params:
`"--twopassMode Basic --twopass1readsN -1 --sjdbOverhang 149 --sjdbGTFtagExonParentGene gene_name --outSAMtype BAM Unsorted --quantMode GeneCounts --outFilterMultimapNmax 200"`

Alignment sorting

Alignments were sorted by coordinate using Picard (*Picard Tools - By Broad Institute*).
Params:
`"VALIDATION_STRINGENCY=LENIENT CREATE_INDEX=true"`

Calculating gene counts

Gene counts were calculated using FeatureCounts from the SubRead package [4].
Params:
`"-p -t exon -g gene_id -s 2 -B -C --minOverlap 60"`

Quality control

Quality control (QC) is performed on raw fastq files using FastQC (*Babraham Bioinformatics - FastQC A Quality Control Tool for High Throughput Sequence Data*). QC based on read alignment was provided by Picard (CollectRnaSeqMetrics, CollectHsMetrics,

CollectAlignmentSummaryMetrics, CollectInsertSizeMetrics, CollectQualityDistributionMetrics, MeanQualityByCycle, CollectBaseDistributionByCycle, CollectGcBiasMetrics, CollectQualityYieldMetrics), RSeQC (Junction Annotation, Junction Saturation, Bam Stat, Read Distribution, Inner Distance, Read GC Content, Read Duplication, Infer Experiment) [5] and STAR. Extensive quality control results are wrapped into a single report using MultiQC [6].

References

1. Mölder F, Jablonski KP, Letcher B, Hall MB, Tomkins-Tinch CH, Sochat V, Forster J, Lee S, Twardziok SO, Kanitz A, Wilm A, Holtgrewe M, Rahmann S, Nahnsen S & Köster J (2021) Sustainable data analysis with Snakemake. *F1000Res* 18, 1-29
2. Martin, M (2011) Cutadapt removes adapter sequences from high-throughput sequencing reads. *EMBnet.journal* 17, 10-12
3. Dobin A, Davis CA, Schlesinger F, Drenkow J, Zaleski C, Jha S, Batut P, Chaisson M & Gingeras TR (2013) STAR: ultrafast universal RNA-seq aligner. *Bioinformatics* 29, 15–21
4. Liao Y, Smyth GK & Shi W (2014) featureCounts: an efficient general purpose program for assigning sequence reads to genomic features. *Bioinformatics* 30, 923–930
5. Wang L, Wang S & Li W (2012) RSeQC: quality control of RNA-seq experiments. *Bioinformatics* 28, 2184–2185
6. Ewels P, Magnusson M, Lundin S & Käller M (2016) MultiQC: summarize analysis results for multiple tools and samples in a single report. *Bioinformatics* 32, 3047–3048
