# Supplementary material for: Data‐driven discovery of gene expression markers distinguishing pediatric acute lymphoblastic leukemia subtypes
Source: Mol Oncol. 2025 Aug 11;19(12):3548–77. doi: 10.1002/1878-0261.70046 (PMC12688183; doi:10.1002/1878-0261.70046)
Supplement: Supplementary file 19 — Text S4. Gene expression correlation analysis. [file MOL2-19-3548-s002.pdf]

Supplementary Text S4 - Analysis of correlation expression

Results

The complete results of the following analyses are available on GitHub (https://github.com/ELELAB/ALL_markers). In B-ALL, CEMiTool identified 3 gene modules of 204 (M1), 179 (M2) and 46 (M3) genes respectively; among our marker genes, one of them (EBF1) belonged to cluster M1, while four of the remaining (RN7SKP80, RN7SKP255, RN7SKP48, SNORA73B) belonged to M2. In T-ALL, the analysis unveiled five gene clusters (M1 to M5), of 131, 113, 73, 73, 34 genes respectively. In this case, only a marker gene was identified as part of a module (RN7SKP255 in M5).

The fact that in some cases the identified genes are part of larger expression correlation networks suggests that they might be involved in overall larger pathways or regulatory networks. We therefore performed gene enrichment analysis on the set of the identified genes from each expression module, focusing on GO Biological Process annotations, to understand whether each module was enriched in genes belonging to specific pathways. We used the same methodology as described in the main text for the remainder of the enrichment analyses performed therein.

It should be noted that only EBF1 was found to be annotated with GO terms in the final output table, possibly because of limitations in the GO annotation itself.

The 15 top enriched terms as per adjusted P-value for B-ALL modules M1 and M2 are shown in Supplementary Figure S9 and S10 below. The most statistically significant enriched GO terms in M1 are connected to immunity-related functions and apoptosis, while M2 is particularly enriched with terms related to protein production (translation) and cellular localization, which are also important for immunity-related functions.

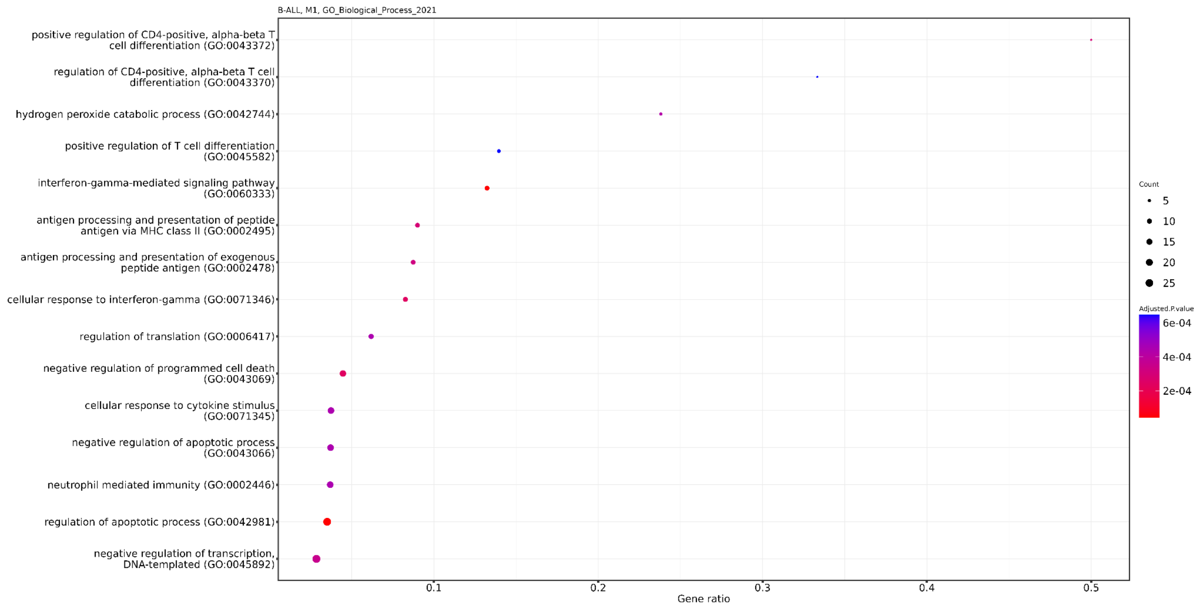

Figure S9: enriched terms in genes belonging to B-cell acute lymphoblastic leukemia (B-ALL) expression correlation module M1.

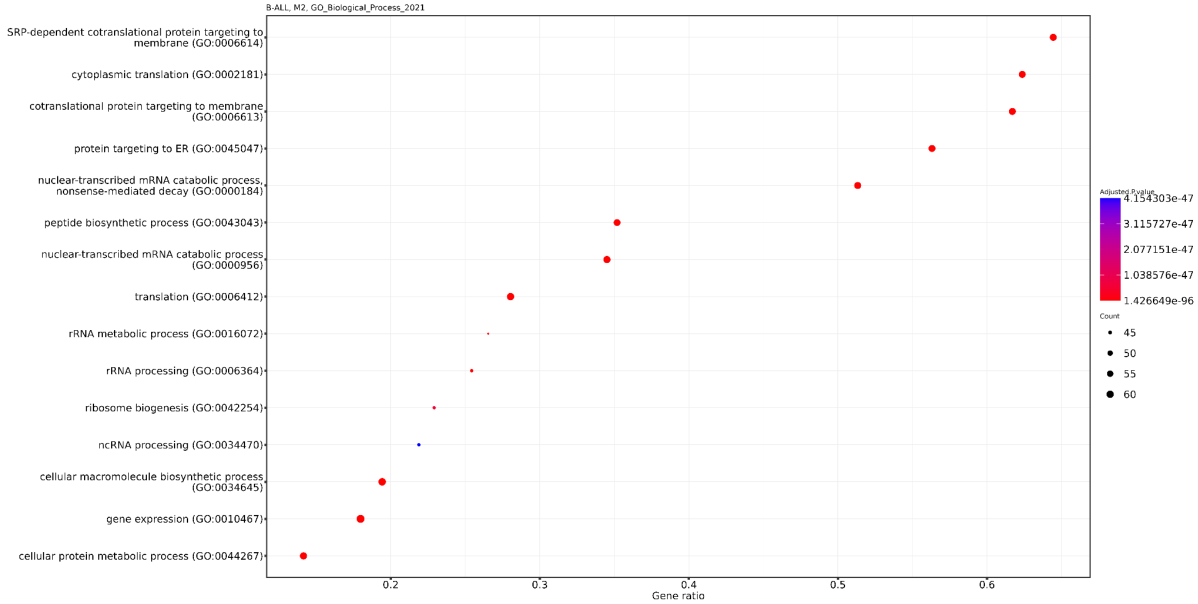

Figure S10: enriched terms in genes belonging to B-cell acute lymphoblastic leukemia (B-ALL) expression correlation module M2.

For T-ALL, the M5 module has only 7 significantly enriched terms (adjusted p-value < 0.05) that are more heterogeneous than the previous case, and include functions connected to leukocyte adhesion and movement (Figure S11).

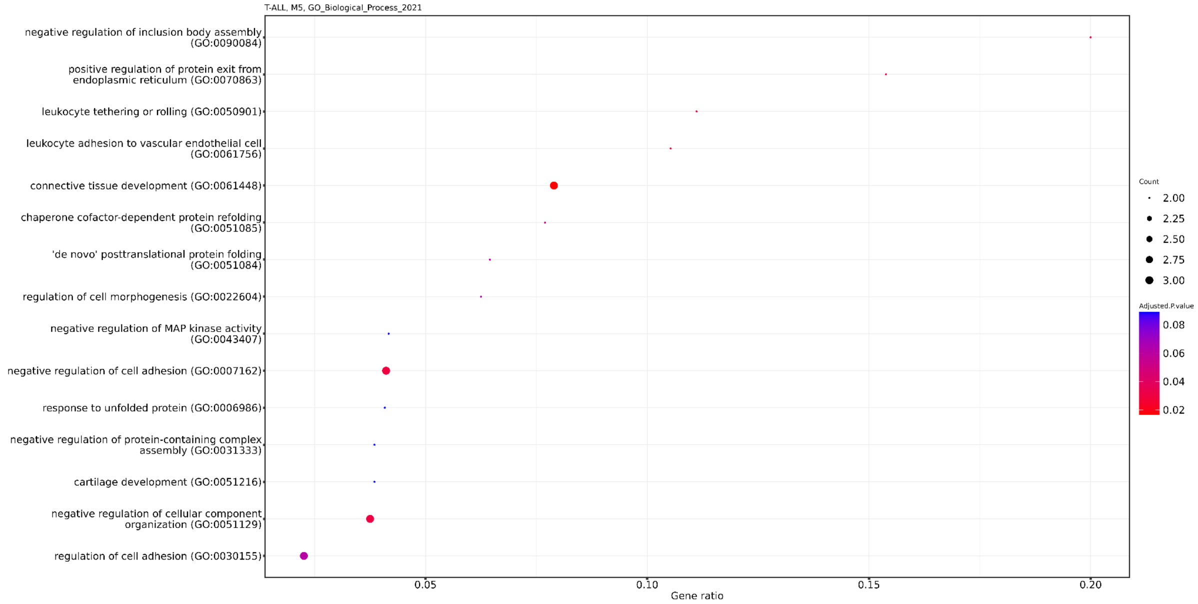

Figure S11: enriched terms in genes belonging to T-cell acute lymphoblastic leukemia (T-ALL) expression correlation module M5.

The fact that some of our markers were found to belong to functionally relevant correlation networks suggests that they are representatives of important cellular functions and that, possibly, other genes belonging to similar functions could be used for the same purpose.
