# Supplementary material for: Data‐driven discovery of gene expression markers distinguishing pediatric acute lymphoblastic leukemia subtypes
Source: Mol Oncol. 2025 Aug 11;19(12):3548–77. doi: 10.1002/1878-0261.70046 (PMC12688183; doi:10.1002/1878-0261.70046)
Supplement: Supplementary file 18 — Text S3. Characteristic direction analysis. [file MOL2-19-3548-s014.pdf]

Supplementary Text S3 - Characteristic direction analysis

Results

From the characteristic direction analysis, we found 1826 significant ENSEMBL gene IDs. All of our predicted markers in our final 14 marker set were also found to be significant from the characteristic direction analysis (**Figure S12**). Additionally, the intersection between the four analyses (characteristic direction analysis, consensus differential expression analysis, elastic net logistic regression and principal component analysis) contained all and only the previously identified 14 markers (**Figure S12**). Thus, the characteristic direction analysis did not change our predicted 14 marker set.

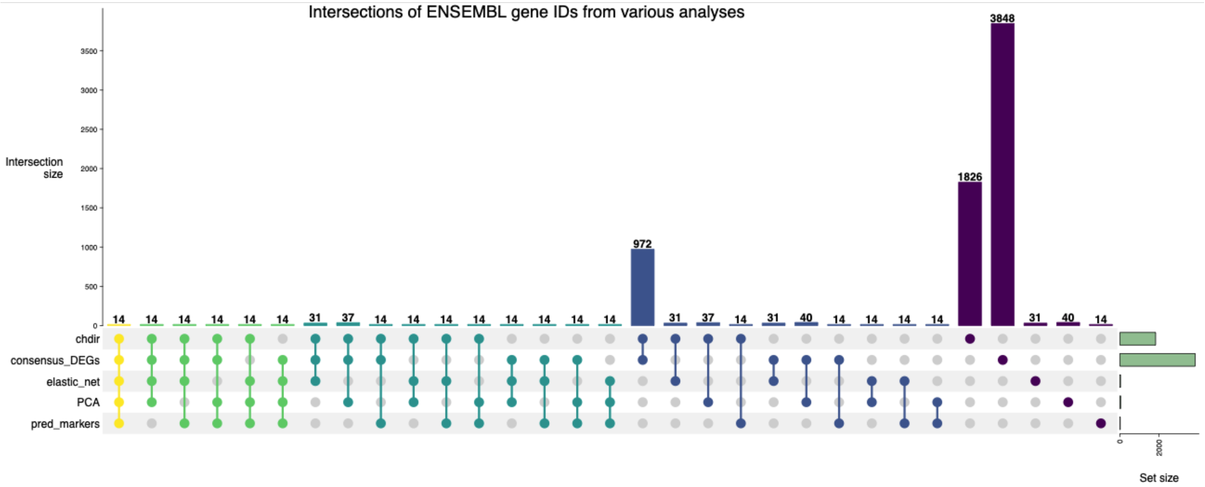

Figure S12. Intersections of ENSEMBL gene IDs from characteristic direction analysis (chdir), consensus differentially expressed genes (consensus_DEGs), elastic net logistic regression (elastic_net), principal component analysis (PCA) and our 14 predicted marker set (pred_markers).
